# Ba–Sr–V as Geogenic and Traffic Tracers in Paediatric Hair from Urban–Industrial Spain, with Co-Located Topsoil Vanadium

**DOI:** 10.3390/toxics14030268

**Published:** 2026-03-19

**Authors:** Antonio Peña-Fernández, Roberto Valiente, Manuel Higueras, Rafael Moreno-Gómez-Toledano, M. Carmen Lobo-Bedmar

**Affiliations:** 1 Area of Legal and Forensic Medicine, Department of Surgery, Medical and Social Sciences, Faculty of Medicine and Health Sciences, University of Alcalá, Ctra. Madrid-Barcelona, Km. 33.600, 28871 Alcalá de Henares, Madrid, Spain; 2 Scientific Computation Research Institute (SCRIUR), Universidad de La Rioja, 26006 Logroño, La Rioja, Spain; 3 Department of Geology, Geography and Environment, Faculty of Philosophy and Letters, University of Alcalá, Calle Colegios 2, 28801 Alcalá de Henares, Madrid, Spain; 4 Area of Human Anatomy and Embryology, Faculty of Medicine and Health Sciences, University of Alcalá, Ctra. Madrid-Barcelona, Km. 33.600, 28871 Alcalá de Henares, Madrid, Spain; 5 Departamento de Investigación Agroambiental, Madrid Institute for Rural, Agricultural and Food Research and Development (IMIDRA), Finca el Encín, Crta. Madrid-Barcelona Km, 38.2, 28800 Alcalá de Henares, Madrid, Spain

**Keywords:** barium, strontium, vanadium, children, geogenic background, biomonitoring, source attribution

## Abstract

Urban–industrial environments can generate mixed geogenic and traffic-related metal signatures in paediatric scalp hair, yet interpretation is challenged by left-censoring and limited health-based guidance values for hair. We quantified barium (Ba), strontium (Sr) and vanadium (V) in archived scalp hair collected in 2001 from children (6–9 years, *n* = 120) and adolescents (13–16 years, *n* = 97) residing in Alcalá de Henares (central Spain). Samples were washed, digested and quantified by Inductively coupled plasma mass spectrometry (ICP–MS; laboratory processing in 2025); results below the limit of detection (LoD) were treated as left-censored using NADA2 (no substitution). In children, Ba and Sr were frequently quantifiable (medians 0.193 and 0.412 µg/g; 38.3% and 23.3% <LoD), whereas V was heavily censored (74.2% <LoD; median 0.003 µg/g). Adolescents showed higher Ba and Sr and broader upper tails (Ba median 0.287 µg/g, P95 2.061 µg/g; Sr median 1.105 µg/g, P95 4.995 µg/g), while V remained low (median 0.011 µg/g, P95 0.052 µg/g). Ba and Sr displayed strong spatial gradients across four residential zones in adolescents (censored-data Peto–Peto tests *p* < 1 × 10^−8^), but V did not (*p* = 0.162). Co-located residential topsoils were available only for V and showed limited between-zone contrast; soil–hair correspondence was weak overall but moderate in adolescent girls (Spearman *ρ* = 0.433). These findings provide a historical baseline and support a cautious tracer-oriented interpretation in which the observed Ba–Sr spatial patterning is consistent with heterogeneous contact with dust- and traffic-influenced surface materials, while V appears less discriminatory in low-contrast community settings.

## 1. Introduction

### 1.1. Paediatric Metal Exposure in Urban–Industrial Environments

Children and adolescents are especially relevant populations in environmental exposure assessment because behavioural, physiological, and developmental factors can modify both contact with contaminants and their biological handling [1,2,3]. In urban–industrial environments, paediatric exposure to trace elements may arise through multiple pathways, including contact with contaminated soils and road dust, inhalation of resuspended particles, and dietary intake, often with marked spatial heterogeneity within the same municipality [4,5]. Integrated biomonitoring studies have shown that these mixed exposure settings can generate measurable spatial patterns in paediatric internal or external biomarkers, particularly when environmental and social determinants coexist [6].

Accordingly, approaches combining environmental matrices with human biomonitoring are increasingly used to improve inference on exposure sources and pathways [3,7]. In Spain, paediatric biomonitoring has expanded mainly through urine-based studies, with selected applications in blood and other matrices, showing that elements such as Ba and V can vary with environmental context, residential setting, and diet [8,9,10,11]. Although urine and blood are generally preferred for short- and medium-term internal dose depending on the element, complementary matrices remain useful where longer integration windows, retrospective assessment, or source-oriented interpretation is needed [3]. Within this framework, scalp hair continues to attract interest because it is non-invasive, easy to store, and potentially informative when decontamination and analytical quality control are carefully standardised [12,13].

### 1.2. Why Focus on Barium, Strontium, and Vanadium as Environmental Tracers?

Barium (Ba) and strontium (Sr) are relevant in urban geochemical studies because they commonly reflect crustal and mineral-dust inputs, yet can also respond to the redistribution of fine particulate material along roads and other trafficked surfaces [4,5]. This makes them potentially informative for evaluating intra-urban gradients where geogenic background and traffic-related resuspension overlap. Evidence from road dust, roadside soils, and related surface materials supports the use of Ba and Sr as tracers of conservative particulate reservoirs influenced by both lithology and non-exhaust traffic processes [14,15].

Interpretation of Ba and Sr in Spain also benefits from continental and Iberian geochemical baselines. The GEMAS (Geochemical Mapping of Agricultural and Grazing land Soil) programme provides harmonised reference distributions for European agricultural and grazing soils [16], and the Iberian reinterpretation is particularly relevant here because Spain contributes most of the grid cells included in that regional compilation [17]. These data confirm that both Ba and Sr are strongly conditioned by lithology and soil chemistry at broad spatial scale, providing an important backdrop for interpreting local urban variation [17].

Vanadium (V) complements Ba and Sr because it represents a different tracer logic. In urban environments, V is often discussed in relation to combustion and industrial activity, but its interpretation is context-dependent because environmental levels are also influenced by parent material and by physicochemical controls on soil retention and mobility [18,19,20]. In Alcalá de Henares, V has previously been measured in urban and industrial soils and showed setting-dependent differences with seasonal variability [21]. Iberian-scale data further indicate that V is strongly associated with ferromagnesian geochemical background, reinforcing that local interpretation must consider both natural and anthropogenic controls [17].

In biomonitoring datasets, V often shows lower detectability and weaker spatial discrimination than Ba and Sr when concentrations are close to analytical limits, which may reduce interpretability at intra-urban scale [3]. For this reason, the Ba–Sr–V combination provides a compact but informative panel: Ba and Sr are useful for evaluating conservative particulate redistribution and dust-related spatial patterning, whereas V offers a more context-dependent indicator of combustion- or industry-related influence under stronger geochemical control [22,23,24].

### 1.3. Hair as a Biomonitoring Matrix for Source-Oriented Studies

Scalp hair is a practical matrix for retrospective biomonitoring because it can be collected non-invasively, stored with minimal burden, and used to reconstruct historical exposure patterns when archived samples are available [12]. These advantages are particularly relevant in source-oriented or population-level studies, where the objective is to evaluate contrasts across age groups, sex, or residential settings rather than to infer individual clinical status.

At the same time, hair concentrations must be interpreted cautiously because they can reflect both endogenous incorporation during hair formation and exogenous deposition of airborne particles or dust onto the hair surface [25]. As a result, interpretation depends on standardised washing/decontamination procedures, robust analytical quality control, and an appropriate statistical treatment of data near detection limits [3,12,25]. Hair is therefore most informative when used as part of an integrated exposure framework and when paired, where possible, with environmental matrices that strengthen source plausibility.

This general approach is supported by previous paediatric studies. In Spain, hair-based datasets have reported age- and sex-related variation and provided reference values for elements including Ba and Sr [26,27]. Other European studies have similarly shown that paediatric hair profiles can discriminate environmental contexts when sampling, analytical methods, and statistical treatment are applied consistently [28]. Together, these studies support the use of hair as a useful matrix for historical baseline definition and for cautious source-oriented interpretation.

### 1.4. Study Rationale, Novelty, and Objectives

Despite increasing interest in hair biomonitoring, paired evidence linking paediatric hair concentrations to contemporaneous environmental matrices remains limited in urban–industrial settings, particularly for historical cohorts. Archived biospecimens can provide a valuable baseline from periods preceding later technological, regulatory, and behavioural changes, but their interpretive value depends on transparent analytical treatment and on appropriate environmental context.

In this study, we analyse scalp hair collected in 2001 from children and adolescents living in Alcalá de Henares (Spain), using a compact Ba–Sr–V panel to evaluate historical exposure patterning in a heterogeneous urban–industrial setting. Ba and Sr are considered primarily as conservative indicators of crustal material and traffic-related dust redistribution, whereas V is examined as a more context-dependent tracer under stronger soil control. Matched topsoil measurements are available only for V, allowing direct assessment of V soil–hair correspondence, while interpretation of Ba and Sr relies on their internal coherence, zone-level spatial patterning, and established behaviour in urban surface materials [29].

The specific objectives were to: (a) characterise Ba, Sr, and V concentrations in scalp hair among children and adolescents, including age- and sex-related contrasts; (b) assess intra-urban spatial variability across predefined environmental zones, testing whether Ba and Sr show gradients compatible with dust/traffic redistribution and whether V shows concordant or contrasting spatial patterning; and (c) evaluate V soil–hair correspondence using matched residential topsoil data, explicitly considering the implications of detection frequency and left-censoring for inference.

## 2. Materials and Methods

### 2.1. Study Area and Design

Alcalá de Henares (40°29′ N, 3°22′ W) is a World Heritage city (~200,000 inhabitants) located ~30 km east of Madrid (central Spain). The municipality combines residential neighbourhoods with legacy and active industrial corridors and high-traffic transport infrastructure, creating a heterogeneous urban–industrial setting for investigating spatially structured exposure to re-suspended surface materials. The present work uses archived paediatric scalp-hair specimens collected in 2001, complemented by contemporaneous topsoil information available for vanadium (V) in the same study area [29]. All archived hair specimens (collected April–May 2001) were retrieved from storage and underwent washing, digestion and Inductively Coupled Plasma Mass Spectrometry (ICP–MS) quantification during 2025 under a single Quality Assurance/Quality Control (QA/QC)-controlled analytical workflow, ensuring full analytical comparability across age strata and zones.

### 2.2. Participants, Ethics and Inclusion Criteria

The study comprises two paediatric strata recruited in Alcalá de Henares: children aged 6–9 years (*n* = 120; 70 girls) and adolescents aged 13–16 years (*n* = 97; 68 girls). Participants were enrolled using inclusion criteria intended to reduce major sources of variability in hair elemental composition [30]. In brief, volunteers had naturally dark, untreated hair; reported no hair dyes or chemical straightening; and were not taking regular medication at the time of sampling.

Sampling was conducted during April–May 2001 as part of a municipal biomonitoring programme designed to establish baseline exposure patterning prior to subsequent urban and technological transitions. The investigation adhered to the Declaration of Helsinki. Written informed consent was obtained from parents/legal guardians, and assent was obtained where appropriate. The retrospective analysis and reporting framework were approved by the relevant institutional ethics committee (Comité de Ética de la Investigación y de Experimentación Animal, University of Alcalá; CEI-EA code CEIP/2025/3/089).

### 2.3. Hair Sampling, Handling, and Zoning

Scalp hair was collected from the occipital region, cut as close as possible to the scalp using stainless-steel scissors. Samples were handled with powder-free gloves and stored in clean paper envelopes under dry conditions during archival storage until laboratory processing. Because hair is a keratinised matrix with generally good stability for trace-element determination under appropriate dry storage conditions, the samples were considered suitable for retrospective analysis; however, the long interval between collection and analysis remains an inherent limitation of this historical biomonitoring dataset. Given the generally accepted mean scalp-hair growth rate of ~1 cm/month—while acknowledging substantial inter-individual variability and collection-related uncertainty—proximal hair primarily integrates exposure over the weeks-to-months preceding sampling, and any time-window assignment should be regarded as approximate [31,32,33].

**Figure 1:**
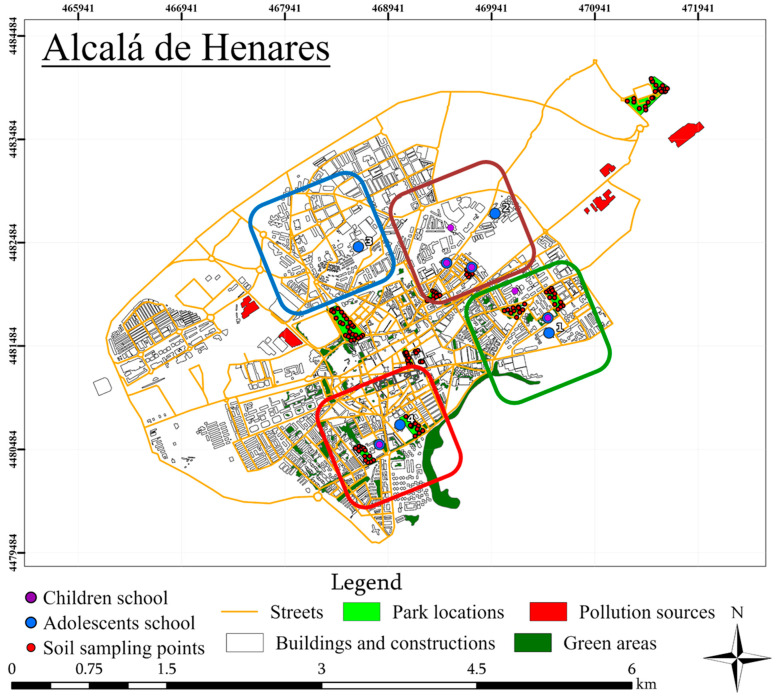
Study area and sampling sites. Children’s primary schools (purple symbols) and adolescents’ secondary schools (blue symbols) are indicated, together with soil sampling locations (red symbols). Where purple and blue symbols overlap, this indicates that the same educational centre includes both a primary school and a secondary school. Coloured outlines delineate the four environmental zones used to evaluate residential setting: Zone I (green outline), low-density housing with abundant green space; Zone II (brown outline), compact urban district with dense residential buildings; Zone III (blue outline), heavy traffic and major roads; and Zone IV (red outline), mixed industrial facilities. Children were recruited from primary schools located in Zones I, II and IV, whereas adolescents were enrolled from secondary schools distributed across all four zones.

To support source-oriented interpretation at the intra-urban scale, the municipality was stratified into four predefined environmental zones following the zoning framework used in related local geochemical/biomonitoring work [29] (Figure 1). In brief, Zone I represents lower-density residential areas with greater green-space availability; Zone II represents a compact urban/residential core; Zone III corresponds to areas dominated by major road traffic; and Zone IV comprises mixed industrial/residential settings. Children were recruited from primary schools located in Zones I, II, and IV, whereas adolescents were enrolled across all four zones, enabling zone-level contrasts in hair Ba–Sr–V distributions.

### 2.4. Dietary Habits (Food-Group Consumption Frequency)

Food-group consumption frequency information was collected to contextualise ingestion as a plausible background exposure pathway for Ba–Sr–V and to evaluate whether broad age- or sex-related dietary shifts could plausibly confound interpretation of hair patterns. Food and drinking water constitute the principal exposure sources for stable Sr in the general population, although their relative contribution varies with hydrogeology and diet [34]. Barium is also encountered via ingestion, with evidence from environmental and total-diet assessments indicating contributions from both diet and drinking water and, in general, higher dietary exposure on a body-weight basis in young children than in adults [35]. Thus, age-group dietary intakes of Sr and Ba have been estimated in Spanish total-diet studies, supporting ingestion as a background pathway in children and adolescents [36]. By contrast, V intake from typical diets is generally low—commonly in the order of tens of micrograms per day—such that, in low-contrast community settings, hair V tends to be most informative as a context-dependent tracer where combustion/industrial signals are strong and environmental contrasts are pronounced [37,38].

Therefore, habitual diet in 2001 was assessed using a brief food-group food frequency questionnaire (FFQ) implemented as a pragmatic screening tool for paediatric field studies. The FFQ was adapted from Spanish dietary assessment practice, drawing on established FFQ design frameworks [39] and on approaches used in contemporaneous Spanish biomonitoring and dietary intake estimation studies in children [40,41]. The instrument covered major food groups (red meat, pork, poultry, processed meats, fish, vegetables, rice/pasta, legumes, bread, fruit, milk, yoghurt, eggs and canned foods). Usual consumption frequency was recorded as never, <2 times/week, 3–4 times/week or ≥5 times/week; missing or insufficiently specified responses were coded as not reported (NR). A food-group screener of this type is also broadly consistent with Spanish dietary habit surveys from the same period, although many population surveys used repeated 24 h recalls rather than short FFQs [42].

For the present manuscript, FFQ data were available only as sex- and age-stratified aggregated frequency distributions (i.e., the percentage of participants in each consumption category for each food group), rather than as participant-level records, and were not available by residential zone. Consequently, food-group consumption frequency was used descriptively to characterise background dietary patterns and plausible ingestion pathways; it could not be used to estimate element-specific intakes (no portion sizes or food-composition linkage), to evaluate dietary determinants at the individual level, or to test whether diet explained the observed spatial gradients in hair Ba–Sr–V.

To provide a concise indicator of habitual intake, a high-frequency metric was derived as % ≥3 times/week (excluding NR) by combining the 3–4/week and ≥5/week categories and re-scaling to exclude not-reported (NR) responses for each item: % ≥3/week (excluding NR) = [( % 3–4/week + % ≥ 5/week )/(100 − %NR)] × 100. For exploratory assessment of between-group differences in consumption frequency, responses were conservatively collapsed to ≥3/week vs. <3/week (excluding NR) and compared across the four age–sex groups using Pearson’s chi-square tests [43]. To account for multiple comparisons across food groups, *p*-values were adjusted using the Benjamini–Hochberg false discovery rate (FDR) procedure, and FDR-adjusted *p*-values (*q*-values) are reported.

### 2.5. Hair Decontamination, Digestion, and Elemental Determination

Given the potential contribution of exogenous particle deposition to hair elemental content, samples were washed using a standardised washing procedure designed to minimise external contamination while preserving endogenous incorporation signals [30,44]. Each bulk hair sample (~1 g) was immersed in 1% (*v*/*v*) Triton X-100 solution (Sigma-Aldrich, St Louis, MO, USA) prepared with ultrapure Milli-Q water (Milli-Q^®^ Direct 8, Merck Millipore, Darmstadt, Germany; resistivity 18.2 MΩ cm), and sonicated in an ultrasonic bath (J.P. Selecta Ultrasons, Abrera, Barcelona, Spain) for five minutes, and the wash step was repeated four times. Specimens were then rinsed three times with ultrapure water and sonicated for an additional five minutes in ultrapure water alone. Cleaned hair was placed on acid-cleaned filter paper and oven-dried at 50 °C to constant weight.

For digestion, approximately 100 mg of the cleaned and dried hair was transferred into acid-washed polytetrafluoroethylene digestion tubes and treated with 2 mL of 65% HNO_3_ (Suprapur^®^, Merck, Darmstadt, Germany). Samples underwent a pre-oxidation step for 12 h, followed by heating at 96 °C for 12 h on a programmable digestion block. Digests were quantitatively brought to 10 mL with ultrapure water, transferred to nitric-acid-washed polypropylene tubes, and stored at −80 °C until analysis. Procedural blanks were included routinely (one blank per five samples). Before instrumental analysis, frozen digest solutions were allowed to thaw at room temperature overnight under laboratory conditions; no external heating was applied.

Barium, Sr, and V were determined in hair digests using inductively coupled plasma–mass spectrometry (ICP–MS) on a PerkinElmer NexION 350D (Waltham, MA, USA). Measurements were performed in helium collision mode to attenuate polyatomic interferences and improve robustness for trace-level quantification in a keratinised matrix.

Following digestion, solutions were diluted 1:5 with ultrapure Milli-Q^®^ water acidified to 1% HNO_3_ (*v*/*v*). External calibration employed a matrix-matched blank and multi-point calibration standards prepared from certified TraceCERT^®^ solutions (Merck, Darmstadt, Germany) in the same acid medium. Niobium was introduced continuously as the sole internal standard to correct for instrumental drift and matrix-related signal variability. Each digest was analysed in duplicate, with three replicate readings per sample and an integration time of 1 s per monitored isotope. The isotopes used for quantification were ^138^Ba, ^88^Sr, and ^51^V.

QA/QC included procedural blanks, calibration-verification standards, duplicate digest analysis, and certified reference materials (CRMs). Analytical performance was verified using two CRMs. First, the NCS DC 73347 human-hair CRM (National Research Centre for Certified Reference Materials, Beijing, China) was analysed every five samples as an independent control for keratinised matrices; certified concentrations (µg g^−1^) were Ba = 17 ± 2 and Sr = 24 ± 1. Second, V was verified using the SPS-SW2 surface-water CRM (Spectrapure Standards AS, Oslo, Norway; batch 127), with a certified concentration of 50 ± 0.3 ng mL^−1^ at 20 °C. Recoveries for certified constituents were within 90–95% of stated values. Procedural blanks and verification standards were measured at the beginning and end of each analytical sequence.

Analytical limits of detection (LoDs) were defined as 3*σ* of blank signals and calculated separately for children and adolescents. Minor LoD differences between groups were attributed to differences in available hair mass after washing and trimming and the consequent dilution requirements rather than to changes in instrumental performance. Under these standardised decontamination and QA/QC-controlled conditions, the resulting dataset provides a defensible historical baseline for Ba–Sr–V concentrations in paediatric hair prior to later shifts in urban exposure profiles.

### 2.6. Vanadium in Topsoils and Soil–Hair Correspondence

Matched environmental measurements were available only for V, based on contemporaneous residential topsoils collected in the Alcalá de Henares study area during the 2001 campaign and linked to participants with co-located sampling [29]. The residential topsoil V values represent an operationally defined pseudo-total fraction obtained by hot nitric-acid digestion (HNO_3_; closed Teflon vessels/bomb digestion) followed by ICP–MS determination in 0–3 cm surface soils, with routine QA/QC including blanks, duplicates and reference material checks [29]. Accordingly, these values are not directly comparable to GEMAS aqua-regia extractables and are used here as a within-area environmental anchor contemporaneous with hair sampling to support tracer-based interpretation of hair V in a heterogeneous urban–industrial setting.

For participants with paired observations, soil–hair correspondence was evaluated using Spearman’s rank correlation coefficient (*ρ*) between hair V concentrations and the corresponding topsoil V levels. Spearman’s ρ was selected because environmental concentration data are typically right-skewed and may deviate from normality, and because the method is robust to monotonic non-linear relationships and the influence of extreme values; statistical significance was assessed at *α* = 0.05. No paired topsoil measurements were available for barium (Ba) or strontium (Sr); accordingly, interpretation of Ba and Sr in the present study cannot be anchored to matched environmental measurements and is therefore restricted to (i) their internal coherence within the hair matrix, (ii) zone-level spatial patterning, and (iii) established tracer behaviour in urban surface materials reported in the wider literature. Any source-oriented inference for Ba and Sr should therefore be regarded as cautious and hypothesis-generating rather than demonstrative. Underlying individual-level concentration data were compiled from the archived dataset underlying prior reporting [29] and re-analysed using the NADA2 package in R, treating values reported as <LoD as left-censored observations (without substitution). Distributional summaries and inferential tests were re-estimated under this harmonised framework to ensure internal consistency across age strata and downstream analyses, including soil–hair association testing for V.

### 2.7. Statistical Analysis and Screening Contextualisation Using Reference Intervals

#### 2.7.1. Descriptive Statistics and Treatment of Left-Censored Data

Elemental concentrations below the analytical limit of detection (LoD) were treated as left-censored observations (<LoD) rather than replaced by substitution values (e.g., LoD/2), consistent with established recommendations for environmental and biomonitoring datasets containing nondetects [24,45]. The extent of censoring (proportion <LoD) and the LoDs applied to each age stratum are reported alongside the descriptive summaries in Section 3. Distributional summaries were estimated using censored-data methods selected according to censoring intensity and sample size. When censoring was <50%, central tendency and spread were obtained using the Kaplan–Meier (KM) nonparametric estimator. For censoring in the 50–80% range, we applied regression on order statistics (ROS) or maximum likelihood estimation (MLE) within a parametric framework when appropriate, following standard practice for moderately to heavily censored concentration data [24,46]. For variables with >80% censoring, we avoided unstable full-distribution estimation and instead emphasised detection frequency and robust upper-tail descriptors where estimable.

Percentiles (including P95) and their confidence bounds were estimated using censoring-aware procedures consistent with the above framework. To support transparent reporting under left-censoring, we report the Kaplan–Meier (KM) mean, i.e., the non-parametric KM estimate of the arithmetic mean that explicitly incorporates <LoD observations as censored values rather than substituted numbers; where indicated, we additionally report the lower 95% confidence limit (LCL) of the KM mean as a conservative bound on the mean under censoring and skewness [24,47,48].

To characterise upper-background levels in this population, reference limits were derived separately for children and adolescents (and by sex where sample size allowed) by estimating the P95 and its 95% confidence interval (CI-P95). Throughout the manuscript, the term “reference interval” is used operationally to denote these upper population reference limits (P95 with confidence bounds), aligned with established IUPAC reference-interval concepts for population benchmarking [49].

Analyses were conducted in R (v4.4.3) using NADA2 for censored-data estimation and inference and supporting packages for standard summaries and diagnostics [50,51].

#### 2.7.2. Group and Spatial Comparisons

Differences by sex and age group (children vs. adolescents) were evaluated using Welch’s t-test or the Wilcoxon rank-sum test, depending on distributional diagnostics and variance homogeneity. Spatial differences among predefined residential zones were tested using methods aligned with censoring status. For variables containing censored values, zone-level summaries were obtained within the KM framework, and an overall Peto–Peto (modified Gehan–Wilcoxon) test was used for one-factor comparisons across zones [47,52].

Where an overall zone effect was detected, post hoc pairwise comparisons were conducted within the same censored-data framework. For fully uncensored variables, distributional assumptions were formally evaluated before test selection using the Shapiro–Wilk test for normality and the Fligner–Killeen test for homoscedasticity; ANOVA-based procedures were applied when assumptions were acceptable, whereas Welch-type methods were used under heteroscedasticity and rank-based non-parametric tests under non-normality. To control multiplicity across zone contrasts (and across the element panel), *p*-values were adjusted using the Benjamini–Hochberg false discovery rate procedure.

#### 2.7.3. Screening-Level Contextualisation Using External Paediatric Hair Reference Distributions (Non-Health-Based)

Because widely accepted health-based biomonitoring guidance values (e.g., biomonitoring equivalents, HBM-I) are generally established for blood and urine and are not routinely available or harmonised for hair for Ba–Sr–V, the present analysis implements screening-level contextualisation rather than a health-threshold hazard quotient [53]. Accordingly, hair concentrations were interpreted against external paediatric hair reference distributions derived from studies reporting percentile statistics and/or formally constructed reference ranges.

As primary comparators within Spain, we prioritised paediatric studies that used ICP-MS and provided distributional detail suitable for benchmarking. In Madrid, Llorente Ballesteros et al. [26] quantified Ba and Sr (among other elements) in hair from 648 healthy participants aged 0–18 years (sampling April 2008–December 2009) and derived 0.95 coverage intervals with 0.95 confidence intervals in line with IUPAC recommendations; this study also reported element-specific proportions below LoD and documented age/sex patterning relevant for interpretation.

For V contextualisation, we additionally considered the large paediatric dataset from Elche (Alicante, eastern Spain) reported by Ruiz et al. [27]. This was an observational, descriptive, cross-sectional study in a Mediterranean setting described as free of excessive pollution problems, analysing 419 hair samples from children aged 3–12 years. Participants were selected by simple random sampling from eight primary schools across municipal districts incorporating urban, rural, and industrial areas.

#### 2.7.4. Exceedance Ratio (ER) for Cross-Population Screening Comparisons

To support transparent, non-health-based benchmarking, we computed an Exceedance Ratio (ER) as a dimensionless descriptor comparing the upper tail of the Alcalá 2001 distribution with an external paediatric comparator distribution:

ER = P95*_study_*/P95*_reference_*

where P95*_study_* is the study-specific 95th percentile and P95*_reference_* is the comparator 95th percentile (or, where reported, the upper limit of a 0.95 IUPAC coverage interval). ER values > 1 indicate that the study upper tail exceeds the external reference level. ER is presented strictly as a screening contextualisation metric, not as a toxicological risk quotient.

## 3. Results

Table 1 and Table 2 summarise detection frequencies and descriptive statistics (LoDs, medians, IQRs and P95 values) for Ba, Sr and V in children and adolescents, overall and stratified by sex. For children, V is available in two derived fields: (i) a legacy V field (V^†^) obtained from the previously processed dataset [29], in which values reported as <LoD were handled by conventional substitution (LoD/2) during manual processing; and (ii) a harmonised V field (V) re-processed in the present study using censored-data methods (NADA2), treating <LoD observations as explicitly left-censored. Accordingly, all children’s V results reported and interpreted in this manuscript (unless explicitly labelled V^†^) refer to the harmonised NADA2 field, ensuring direct comparability with adolescent V summaries and with all zone- and correlation-based analyses involving V.

Although LoD/2 substitution has been widely used in environmental chemistry, it can bias distributional descriptors and hypothesis tests when censoring is high [45,54]. This is particularly relevant when censoring affects the lower tail, where percentiles and dispersion metrics can become unstable under substitution-based handling [55]. Moreover, once <LoD values are replaced by numeric substitutes, the original nondetect flag can be partially or fully lost in exported datasets; when censoring indicators are later reconstructed (e.g., due to rounding and borderline values near the detection boundary), the number of observations classified as <LoD may differ slightly between workflows. To address these limitations and harmonise inference across age strata, we have re-processed children’s V using statistically robust methods for left-censored data [24], implemented in the NADA2 framework [48]. Any divergence between V^†^ and harmonised V is therefore expected to be most pronounced under heavy censoring and reflects the sensitivity of substitution-based summaries (and reconstructed <LoD counts) to handling at the detection boundary, rather than differences in the underlying analytical measurements.

### 3.1. Detection Frequencies and Concentrations in Children

In children, Ba and Sr were quantified in the majority of samples, whereas V showed substantial left-censoring (74.2% of observations <LoD; *n* = 31; LoD 0.02 µg/g). Barium was detected in 74 samples (38.3% <LoD; LoD 0.124 µg/g), with a median of 0.193 µg/g (IQR 0.124–0.350) and a P95 of 0.629 µg/g. Strontium was detected in 92 samples (23.3% <LoD; LoD 0.187 µg/g), with a median of 0.412 µg/g (IQR 0.195–0.894) and a P95 of 2.155 µg/g. For vanadium, the median was 0.003 µg/g (IQR 0.0004–0.0235) and the P95 was 0.428 µg/g (Table 1).

Sex-stratified distributions indicated higher Ba and Sr in girls than boys. The median Ba was 0.108 µg/g in boys versus 0.271 µg/g in girls (*p*-value = 0.000004), and the median Sr was 0.197 µg/g in boys versus 0.688 µg/g in girls (*p*-value = 6.78 × 10^−10^). For V, a modest sex contrast was observed (*p*-value = 0.024), with higher values in boys (median 0.013 µg/g; 64.0% <LoD) than girls (81.4% <LoD); however, inference for girls was constrained by heavy censoring.

In children, bivariate correlation analysis of hair elemental profiles showed a consistent and statistically robust co-variation between Sr and Ba. Specifically, hair Sr was positively associated with hair Ba in the overall children dataset (Spearman’s *ρ* = 0.71; *p* < 0.01), indicating that higher Sr concentrations tended to occur alongside higher Ba concentrations. This pattern remained evident after sex stratification, with a positive Sr–Ba association observed both in boys (*ρ* = 0.50; *p* < 0.01) and in girls (*ρ* = 0.74; *p* < 0.01). Collectively, these findings could support a coherent Ba–Sr hair signature in children, and the persistence of the association across strata suggests that the relationship is not driven solely by sex-specific variability in exposure or hair matrix characteristics. In contrast, V showed little evidence of co-variation with Ba or Sr in the overall children dataset (V–Ba: *ρ* = 0.06; *p* = 0.517; V–Sr: *ρ* = 0.06; *p* = 0.534). After sex stratification, V remained weakly and non-significantly associated with Ba in both boys (*ρ* = 0.21; *p* = 0.146) and girls (*ρ* = 0.11; *p* = 0.351), while a modest positive V–Sr association was observed in boys only (*ρ* = 0.32; *p* = 0.022) and not in girls (*ρ* = 0.04; *p* = 0.725). Given the substantial left-censoring of V in children, these V-related correlations should be interpreted cautiously as descriptive patterns rather than strong evidence of shared sources or coupled uptake.

### 3.2. Detection Frequencies and Concentrations in Adolescents

In adolescents (13–16 years; Table 2), Ba, Sr, and V were more consistently quantifiable than in children, although censoring remained relevant, particularly for V in males (75.9% of observations <LoD). Barium was detected in 63 samples (35.1% <LoD; LoD 0.153 µg/g) with a median of 0.287 µg/g (IQR 0.153–0.948) and a P95 of 2.061 µg/g. Strontium was detected in 61 samples (37.1% <LoD; LoD 0.434 µg/g) with a median of 1.105 µg/g (IQR 0.434–2.390) and a P95 of 4.995 µg/g. Vanadium was detected in 47 samples (51.5% <LoD; LoD 0.011 µg/g) with a median of 0.011 µg/g (IQR 0.006–0.020) and a P95 of 0.052 µg/g.

In adolescents, sex contrasts were evident for Sr and V but not for Ba, with concentrations significantly higher in females than males for both elements: median Sr was 1.240 µg/g in females versus 0.668 µg/g in males (*p*-value = 0.006), and median V was 0.013 µg/g in females versus 0.001 µg/g in males (*p*-value = 0.004).

In adolescents, correlation analysis of the hair elemental profile identified a highly coherent Sr–Ba signal. Hair Sr was very strongly and positively associated with hair Ba in the overall adolescent dataset (*ρ* = 0.90; *p* < 0.01), indicating that individuals with higher Sr concentrations also tended to present higher Ba concentrations. This relationship persisted after sex stratification and remained extremely strong in boys (*ρ* = 0.98; *p* < 0.01) and similarly strong in girls (*ρ* = 0.91; *p* < 0.01). The magnitude and consistency of these associations across the adolescent sample supports a shared underlying driver for Sr and Ba in hair, compatible with common external inputs and/or parallel incorporation into the hair matrix rather than an association attributable solely to sex-specific variability.

### 3.3. Age Comparison

Across paediatric strata, Ba and Sr were consistently quantifiable and exhibited clear age-related shifts in their distributional profiles (Table 1 and Table 2). Children showed median concentrations of 0.193 µg/g for Ba and 0.412 µg/g for Sr (with 38.3% and 23.3% <LoD, respectively), whereas adolescents displayed higher central tendency and a markedly more elevated upper tail (Ba median 0.287 µg/g; Sr median 1.105 µg/g; P95: 2.061 µg/g for Ba and 4.995 µg/g for Sr). These differences were statistically significant for both elements (*p*-values = 0.0014 and 1.67 × 10^−5^, respectively; Table 2). In contrast, V showed pronounced age-dependent quantifiability (*p*-value = 0.365): children’s hair V was predominantly left-censored (74.2% <LoD), while adolescents had a substantially higher detection frequency (51.5% <LoD) with concentrations centred near the analytical limit (median 0.011 µg/g; IQR 0.006–0.020). Overall, these findings indicate a more prominent Ba–Sr signal in adolescent hair alongside improved interpretability of V relative to children, consistent with age-linked differences in exposure opportunity and microenvironmental contact patterns.

### 3.4. Spatial Contrasts by Residential Zone

Spatial contrasts differed sharply by age stratum. Among children (Table 3), zone-level differences were limited: Ba showed only borderline heterogeneity across Zones I, II and IV (Peto–Peto *p*-value = 0.053), while Sr showed no evidence of a zone effect (*p*-value = 0.736). For V, neither the historical dataset reported by Peña-Fernández [29] (V^†^; NS) nor the present censored-data analysis showed significant zone differences (*p*-value = 0.139). Because children were recruited from Zones I, II and IV only (i.e., Zone III not represented), inference on spatial structure in children is necessarily constrained by the sampling frame. Consistent with the absence of significant overall zone effects, no post hoc pairwise groupings are displayed in Table 3.

In adolescents (Table 4), zone gradients were pronounced for Ba and Sr when zone-level central tendency was estimated using the Kaplan–Meier (KM) mean to accommodate left-censoring. For Ba, the KM mean increased from 0.297 µg/g (Zone I) to 0.537–0.535 µg/g (Zones II–III) and peaked at 1.872 µg/g in Zone IV, with a strong overall zone effect (*p*-value = 2.66 × 10^−18^). The conservative lower confidence limit of the KM mean (LCL Mean) showed the same ordered pattern (0.172 and 0.254 in Zones I–II; 0.352 in Zone III; 1.529 in Zone IV), and FDR-adjusted post hoc comparisons indicated that Zones I and II were comparable, Zone III was higher than Zones I–II, and Zone IV was higher than all other zones (I = II < III < IV).

Strontium exhibited an analogous spatial structure: the KM mean increased from 0.989 µg/g (Zone I) to 1.489 µg/g (Zone II) and 1.865 µg/g (Zone III), reaching 3.832 µg/g in Zone IV (overall *p*-value = 2.61 × 10^−9^). LCL Mean values supported the same monotonic ordering (0.652 and 0.875 in Zones I–II; 1.302 in Zone III; 2.314 in Zone IV), with identical FDR-adjusted post hoc grouping (I = II < III < IV). In contrast, V showed no evidence of spatial separation across adolescent zones (*p*-value = 0.162) despite improved detection relative to children. Collectively, these results demonstrate strong intra-urban spatial structuring for Ba and Sr in adolescents under the zoning framework, whereas V displayed weaker spatial patterning.

### 3.5. Food-Group Consumption Frequency Patterns

Food-frequency questionnaire summaries are presented descriptively to contextualise ingestion as a background exposure pathway (Table S1). Milk and bread were frequently consumed by most participants, with high-frequency milk intake (≥3 times/week, excluding not-reported responses) near-universal in children (boys 100.0%; girls 98.6%) and remaining high in adolescents (boys 97.6%; girls 89.5%). Bread intake was also consistently high (children: boys 86.5%, girls 84.9%; adolescents: boys 95.2%, girls 86.0%). Fruit and yoghurt were commonly reported but showed an age-related shift: high-frequency fruit intake decreased from childhood to adolescence, from 86.2% to 69.0% in boys and from 89.0% to 80.7% in girls; similarly, high-frequency yoghurt intake decreased from 94.8% to 83.3% in boys and from 87.5% to 73.7% in girls.

**Figure 2:**
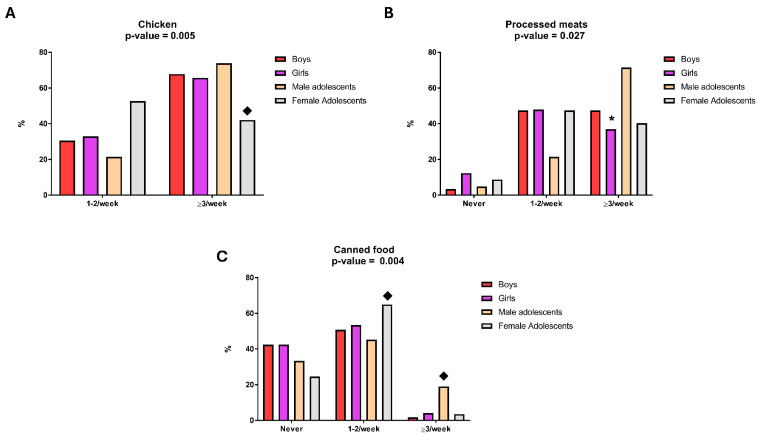
Food-group consumption frequency contrasts highlighted by exploratory *χ*^2^ testing (four age–sex strata). Panels show (**A**) chicken/poultry, (**B**) processed meats, and (**C**) canned foods. Bar charts summarise the distribution of consumption frequency categories across four age–sex strata (children: boys, girls; adolescents: boys, girls), using the frequency groupings reported in Table S1. Between-group differences were evaluated using Pearson’s chi-square (*χ*^2^) tests. For processed meats and canned foods, tests compared 4 strata × 3 response categories (Never, 1–2/week, ≥3/week; df = 6). For chicken/poultry, the “Never” category was absent (zero counts), so the comparison reduced to 4 strata × 2 categories (1–2/week, ≥3/week; df = 3). Multiple testing was controlled using the Benjamini–Hochberg false discovery rate (FDR) across food groups (Table S2). Chicken/poultry (*p* = 0.005; *q* = 0.035) and canned foods (*p* = 0.004; *q* = 0.035) retained statistical significance after FDR correction, whereas processed meats showed a nominal (unadjusted) association (*p* = 0.027) that did not survive FDR adjustment (*q* = 0.126). Symbols indicate statistical status: rhombus denotes FDR-significant contrasts (*q* < 0.05), whereas asterisk denotes nominal significance only (*p* < 0.05 but *q* ≥ 0.05). These patterns are presented as descriptive contextual information on potential ingestion pathways; FFQ data were not available at the individual or zone level and therefore are not used to attribute the observed spatial gradients in hair Ba–Sr–V.

Sex contrasts were evident for selected food groups. Processed meats were reported more frequently in boys than girls in both strata (children: 48.3% vs. 37.9%; adolescents: 73.2% vs. 41.8%), and canned foods were infrequently reported in children (boys 1.8%; girls 4.1%) but more frequent in adolescent boys than girls (19.5% vs. 3.8%). Exploratory comparisons of collapsed consumption frequency (≥3/week vs. <3/week, excluding not reported) across the four age–sex groups identified limited evidence of between-group heterogeneity after multiple-comparison control (Table S2). Following Benjamini–Hochberg FDR adjustment across food groups, only chicken/poultry (*χ*^2^ = 12.952, df = 3, *p*-value = 0.005, *q*-value = 0.035) and canned foods (χ^2^ = 19.371, df = 6, *p*-value = 0.004, *q*-value = 0.035) retained statistical significance. For chicken/poultry, this heterogeneity was primarily driven by lower high-frequency intake among adolescent girls (≥3/week: 44.4%) compared with the other strata (children: 66.6–69.0%; adolescent boys: 77.5%). For canned foods, the signal was driven by markedly higher high-frequency intake in adolescent boys (19.5%) relative to adolescent girls (3.8%) and both child strata (1.8–4.1%). These contrasts are illustrated in Figure 2.

Moreover, several additional items showed nominal (unadjusted) differences (specifically processed meats: *p*-value = 0.027) that did not remain significant after FDR correction (*q*-value = 0.126). Milk consumption also showed a nominal between-group association in the Pearson chi-square test (*p*-value = 0.037); however, this result should be interpreted with caution, as the distribution was highly unbalanced with the vast majority of participants reporting consumption ≥3 times/week and a high proportion of cells with low expected counts, limiting the reliability of the chi-square inference. Given that FFQ data were available only as aggregated age–sex distributions (without individual- or zone-level linkage), consumption frequency patterns are used here for descriptive contextualisation of ingestion pathways rather than as determinants of hair Ba–Sr–V distributions or spatial gradients.

### 3.6. Vanadium in Residential Topsoils and Soil–Hair Correspondence

Contemporaneous residential topsoils were available for V only, enabling environmental contextualisation of hair V against co-located surface materials. Across the four residential zones, topsoil V showed a narrow and highly overlapping distribution [29], with mean (SD) values of 8.90 (3.67) mg/kg in Zone I (range 3.54–17.25; *n* = 24), 9.09 (3.74) mg/kg in Zone II (range 4.11–18.29; *n* = 23), 9.24 (4.31) mg/kg in Zone III (range 2.13–16.71; *n* = 23), and 9.27 (4.42) mg/kg in Zone IV (range 2.44–16.82; *n* = 25). Consistent with the close similarity of these zone-wise summaries, no statistically significant zone differences were observed for topsoil V.

Soil–hair correspondence for V was evaluated using Spearman’s rank correlation (*ρ*) between individual hair V concentrations and the corresponding co-located topsoil V measurements. In children, the overall association was weak and not statistically significant (*ρ* = 0.104; *p* = 0.259; *n* = 120). Sex-stratified analyses suggested heterogeneity: girls showed no evidence of correspondence (*ρ* = −0.054; *p* = 0.660; *n* = 70), whereas boys showed a small but statistically significant positive association (*ρ* = 0.293; *p* = 0.039; *n* = 50). In adolescents, the overall association remained weak and non-significant (*ρ* = 0.102; *p* = 0.318; *n* = 97); sex-stratified analyses suggested heterogeneity: adolescent girls exhibited a moderate positive correspondence (*ρ* = 0.433; *p* = 0.00023; *n* = 68), whereas adolescent boys showed a modest inverse, non-significant association (*ρ* = −0.310; *p* = 0.101; *n* = 29). Given (i) the limited between-zone contrast in topsoil V and (ii) substantial left-censoring of hair V (particularly in children), these correlations should be interpreted cautiously as descriptive indicators of monotonic correspondence under constrained environmental variability, rather than as strong evidence of exposure–biomarker coupling. Additional unmeasured modifiers may have contributed to this sex-specific heterogeneity, including differences in time–activity patterns, contact with non-residential surfaces, hair length, and routine grooming/washing practices, all of which may influence particle deposition and persistence on the hair surface.

### 3.7. Population Reference Limits and Screening-Level Risk Contextualisation

Population reference limits (internal benchmarks) were derived as the 95th percentile (P95) with its confidence interval (CI-P95), estimated separately for children and adolescents and, where sample size allowed, by sex (Table 1 and Table 2). In children, the overall P95 values were 0.629 µg/g for Ba (CI-P95 0.447–0.687), 2.155 µg/g for Sr (CI-P95 1.183–2.509), and 0.428 µg/g for V (CI-P95 0.165–1.109). Sex-specific P95 values in children were 0.485 µg/g (boys) vs. 0.717 µg/g (girls) for Ba, 0.758 µg/g (boys) vs. 2.441 µg/g (girls) for Sr, and 0.567 µg/g (boys) vs. 0.222 µg/g (girls) for V; for girls, the P95 confidence interval was not estimable under high censoring and therefore an additional upper-tail descriptor is reported (P97.5 = 0.328 µg/g).

In adolescents, the overall P95 values were 2.061 µg/g for Ba (CI-P95 1.679–2.668), 4.995 µg/g for Sr (CI-P95 3.454–6.342), and 0.052 µg/g for V (CI-P95 0.037–0.072). Sex-specific P95 values in adolescents were 2.425 µg/g (males) vs. 2.004 µg/g (females) for Ba, 2.786 µg/g (males) vs. 6.023 µg/g (females) for Sr, and 0.057 µg/g (males) vs. 0.053 µg/g (females) for V.

For screening-level contextualisation against external paediatric hair reference distributions (non-health-based), we benchmarked study-specific upper tails (P95) using the exceedance ratio (ER) against two Spanish paediatric comparators. First, we compared our Ba and Sr P95 values with those reported for the Madrid paediatric cohort by Llorente Ballesteros et al. [26], which provides Ba and Sr percentiles and an IUPAC 0.95 coverage interval (upper reference limits: Ba 1.8 µg/g; Sr 6.73 µg/g; reported P95: Ba 1.8 µg/g; Sr 6.74 µg/g). Relative to these Madrid upper-tail values, children were below the Madrid benchmarks for both Ba (ER = 0.35) and Sr (ER = 0.32). Adolescents showed a modestly elevated upper tail for Ba (overall ER = 1.15; males ER = 1.35; females ER = 1.11) while remaining below the Madrid benchmark for Sr (overall ER = 0.74; males ER = 0.41; females ER = 0.89). Vanadium was not reported in the Madrid hair reference study and therefore could not be benchmarked using this comparator.

Second, we compared our P95 values with the Ruiz et al. [27] Spanish reference compilation (Elche, Alicante), using the upper bound of the reported 5th–95th percentile range as a pragmatic external P95 comparator (Ba 0.901 µg/g; Sr 13.37 µg/g; V 0.314 µg/g). Under this comparator: (i) children were below the reference for Ba (ER = 0.70) and Sr (ER = 0.16), while children’s V upper tail was moderately higher overall (ER = 1.36), driven primarily by boys (ER = 1.81) with girls below the comparator (ER = 0.71); (ii) adolescents showed an elevated upper tail for Ba (ER = 2.29 overall; 2.69 in males; 2.23 in females), while remaining below the reference for Sr (ER = 0.37 overall) and V (ER = 0.17 overall). Given the high proportion of <LoD values for V in children, these V comparisons should be interpreted cautiously.

Ruiz et al. [27] also reported conservative single-value screening limits (i.e., simple “flagging” thresholds) proposed for broader use: Ba < 0.9 µg/g, Sr < 5 µg/g, and V < 0.4 µg/g. We therefore performed an additional screening comparison using the same exceedance ratio approach (ER = P95*_study/LimitRuiz_*). Under this criterion, children’s Ba upper tail remained below the screening limit (P95 = 0.629 µg/g; ER = 0.70), and children’s Sr upper tail was also well below the limit (P95 = 2.155 µg/g; ER = 0.43). Children’s V upper tail was close to, and slightly above, the screening limit overall (P95 = 0.428 µg/g; ER = 1.07), with sex-stratified ratios of ER = 1.42 in boys (P95 = 0.567 µg/g) and ER = 0.56 in girls (P95 = 0.222 µg/g); using the girls’ P97.5 (0.328 µg/g) as a sensitivity descriptor yields ER = 1.04 vs. the Ruiz external P95 (0.314 µg/g) and ER = 0.82 vs. the Ruiz screening limit (0.4 µg/g), without changing the qualitative interpretation. For adolescents, the Sr upper tail was essentially at the screening limit overall (P95 = 4.995 µg/g; ER ≈ 1.00) and exceeded the limit among females (P95 = 6.023 µg/g; ER = 1.20). In contrast, adolescent V remained well below the screening limit (P95 = 0.052 µg/g; ER = 0.13). Adolescent Ba exceeded the screening limit in both sexes (overall P95 = 2.061 µg/g; ER = 2.29), indicating an elevated upper tail relative to this conservative benchmark [27].

The proportion of <LoD observations for children’s V differed slightly between the legacy [29] processing and the harmonised NADA2 re-analysis (79.5% vs. 74.2% <LoD). This difference does not reflect a change in the underlying analytical measurements; rather, it is attributable to data handling at the detection boundary in the legacy workflow (LoD/2 substitution and incomplete preservation of the nondetect flag), which can lead to small discrepancies when <LoD indicators are later reconstructed from the processed spreadsheet (e.g., rounding and borderline values near the detection limit). All V summaries and inferential results reported in this manuscript are therefore based on the harmonised censoring indicators and censored-data estimation framework.

## 4. Discussion

### 4.1. Interpretive Context

Hair biomonitoring provides a pragmatic, non-invasive window into paediatric contact with environmental trace elements over weeks-to-months. Nevertheless, interpretation needs to remain source- and screening-oriented because hair concentrations integrate (i) keratin-bound incorporation during fibre formation and (ii) an exogenous particulate fraction associated with deposited and retained dust that may not be fully removed by washing, with well-described limitations in using hair as a biomarker of both toxic and essential elements and with potential external interference from ambient pollution [25,56]. Accordingly, the present study applies conservative decision rules: (a) strong element–element coherence and zone-structured gradients are interpreted primarily as indicators of heterogeneous contact with shared environmental reservoirs (e.g., mineral/road-deposited dust and resuspended surface material), whereas (b) weak spatial contrast under substantial left-censoring is interpreted as limited discriminatory power rather than evidence for the absence of relevant sources. Transparent reporting and interpretation of detection limits and censored distributions are therefore essential for comparability and screening use, consistent with recent minimum information requirements for human biomonitoring studies [57]. Within this interpretive context, sex- and age-related patterning is evaluated against Spanish paediatric evidence and Alcalá distributions are compared with external reference datasets used for screening-style interpretation.

### 4.2. Sex and Age Patterning in Relation to Spanish Paediatric Evidence

Spanish paediatric hair datasets consistently show that sex and age can influence hair element distributions, although effects are element-specific and often secondary to microenvironmental contact and external deposition dynamics [26,27]. In the Alcalá cohort, the sex structure is not uniform across elements or age strata, which is consistent with this Spanish evidence base.

Sex contrasts in paediatric hair metal profiles are frequently reported yet remain element- and context-dependent, reflecting a mixture of biological and behavioural determinants (e.g., hair length and surface area, grooming and washing practices, and particle retention time). For Ba and Sr, the direction of sex contrasts observed in younger children (higher values in girls) is coherent with Spanish reference work in Madrid, which identified sex as an influencing factor for alkaline-earth elements in paediatric hair [26]. Mechanistically, this pattern is plausibly explained by hair- and behaviour-mediated differences that modify the retention of deposited particles on the hair shaft, including grooming and washing regimens and time spent in outdoor microenvironments with resuspended dust [25,58]. Although keratin-bound incorporation cannot be excluded, the balance of evidence indicates that exogenous particulate interference contributes materially to measured hair burdens for several elements and may be differentially expressed across sex groups through hair-care practices and particle–hair contact time [25,56]. This interpretation is also consistent with broader toxicological evidence that sex differences in biomonitoring may arise from differences in exposure opportunity and toxicokinetics, rather than sex-specific sources [59].

In adolescents, sex patterning differs from younger children: Sr and V are higher in females, whereas Ba does not show a consistent separation by sex. This age-stratified behaviour is compatible with (i) the increasing heterogeneity of exposure opportunity during adolescence (greater mobility and broader activity spaces) and (ii) the growing importance of sex-differentiated hair characteristics and grooming practices during puberty and mid-adolescence [59,60]. Importantly, the fact that Sr—an element with strong mineral affinity—shows sex differences in adolescents aligns with European multi-site evidence indicating that adolescent girls can present systematically higher hair Sr than boys, even across differing environmental contexts [60].

For Sr and V, higher concentrations in females—particularly in adolescence—are consistent with patterns described in several hair biomonitoring studies in which females show higher levels for a subset of elements. Mechanistically, this is compatible with longer hair–air/particle contact time and increased opportunity for particle adsorption, rather than a sex-specific source acting uniquely on one group. Recent microchemical evidence indicates that exogenous contamination can measurably affect not only the cuticle but also cortex layers for several elements, including Ba and Sr, reinforcing the need to interpret modest sex contrasts with attention to external deposition pathways [61]. Experimental and field evidence also shows that ambient particulate matter can contaminate hair despite washing, particularly under high-PM scenarios, supporting a conservative interpretation of small sex differences when particle exposure differs by behaviour or microenvironment [25].

Therefore, the Alcalá sex patterning is best interpreted as consistent with established paediatric hair behaviour: sex modifies the expression of hair concentrations for specific elements and age strata, likely through a combination of external deposition dynamics and behavioural/hair-regimen factors, rather than indicating a discrete sex-specific emission source.

Age-related differences in hair element burdens are commonly attributed to developmental changes in mobility, time–activity patterns, and cumulative contact with dust and outdoor surfaces, together with changes in hair properties and personal-care practices across childhood and adolescence. Higher Ba and Sr in adolescents relative to younger children aligns with a dust/mineral-contact interpretation and with reports from European paediatric hair studies in which lithogenic/particle-associated elements tend to increase with age or show stronger upper-tail behaviour in older groups.

Age-related shifts in Alcalá also align with the Spanish paediatric literature. The increase in Ba and Sr from children to adolescents is consistent with a transition towards broader environmental contact and greater cumulative interaction with road-deposited dust and resuspended surface materials [26,27]. This interpretation is strengthened by the well-established role of Ba and Sr as markers of mineral/particle-associated exposure in urban environments and by the methodological evidence that particle contamination can materially influence measured hair burdens for several elements, particularly in the upper tail [25].

For V, age patterning is typically more context-sensitive because V may represent mixed contributions from crustal material and combustion/industrial sources, with local source specificity varying substantially by setting. Accordingly, differences between children and adolescents are interpreted most robustly when supported by concordant spatial gradients or co-variation with other source-indicator elements, rather than treated as a single-source proxy.

In contrast, V in Alcalá behaves as a low-level, context-dependent element: substantial left-censoring in children limits inferential resolution, while improved quantification in adolescents is not accompanied by strong intra-urban structuring. This is consistent with Spanish monitoring in Tarragona schoolchildren, where V in hair has shown low detection frequencies and has sometimes been excluded from inferential analyses due to censoring, even in a setting under industrial scrutiny [62]. Accordingly, V interpretation in younger children should remain conservative and explicitly conditioned on censored-data handling, whereas Ba and Sr provide a more stable and interpretable age-structured signal. Under heavy censoring, apparent subgroup differences may be driven by a small number of detectable values, so robustness should be interpreted conservatively when detection frequency is low.

### 4.3. Diet as Contextual Background and Comparison with Spanish Paediatric Evidence from the Same Period

The FFQ profile in the study cohort—frequent consumption of staple foods (milk and bread) and high reporting of fruit, with lower and more variable reporting for fish and a higher frequency of processed-meat intake in boys—appears broadly consistent with Spanish paediatric dietary descriptions from the late-1990s/early-2000s, including national data from the enKid study (1998–2000) and related analyses of Mediterranean diet adherence. The enKid study was a nationwide, cross-sectional nutrition survey conducted in Spain in 1998–2000 that characterised dietary habits and nutritional status across children, adolescents and young adults (commonly reported as approximately 2–24 years, depending on the analysis) [63]. Within this framework, the KIDMED (Mediterranean Diet Quality Index for children and adolescents) was developed as a 16-item score to summarise adherence to the Mediterranean dietary pattern in young people. In the original KIDMED report derived from the enKid sample, the proportion reporting daily fruit (or fruit juice) consumption was 89.1% among those aged 2–14 years and 84.9% among those aged 15–24 years, while the proportion reporting consumption of a dairy product at breakfast was 85.0% (2–14 years) and 80.9% (15–24 years) [64]. These figures are directionally concordant with the food intake summaries in the present study, in which high-frequency dairy and fruit reporting is common and shows an age-related decline (Table S1). In parallel, the enKid-based paper describing food consumption in the Spanish population aged 2–24 years highlighted relatively high intakes of meat/sausages with comparatively low fish and vegetables [63], which is compatible with the descriptive patterning observed here (notably higher processed-meat frequency in boys and more variable fish reporting). School-age reviews from the same decade similarly portray heterogeneous adherence to fruit/vegetable recommendations against a backdrop of widely consumed staples [65]. Collectively, these comparisons indicate that the food-group intake profile in the present cohort was not atypical for Spanish children/adolescents in that period and, therefore, is unlikely to represent a distinctive dietary position that would plausibly generate pronounced intra-urban gradients in hair Ba–Sr–V.

Notably, exploratory chi-square testing of collapsed consumption frequency across the four age–sex strata identified only limited evidence of heterogeneity after Benjamini–Hochberg false discovery rate (FDR) adjustment: chicken/poultry and canned foods retained significance (*q*-value = 0.035), whereas several nominal associations (e.g., processed meats, milk) did not survive FDR correction. Importantly, these two FDR-retaining signals reflected sex-specific patterning within adolescence rather than a uniform child–adolescent dietary shift (Figure 2): high-frequency chicken/poultry consumption (≥3/week) was markedly lower in adolescent girls than in adolescent boys and both child strata, while high-frequency canned-food consumption was higher in adolescent boys than in the other strata. Because FFQ data were not available at the individual or zone level, these group-level contrasts cannot be used to attribute the pronounced adolescent Ba–Sr spatial gradients to dietary intake and are interpreted only as contextual background.

In this context, diet is best interpreted as a background exposure pathway rather than the primary driver of the observed hair Ba–Sr–V distributions, particularly because FFQ contrasts were modest, largely non-robust after multiplicity control, and—in some items with highly unbalanced response distributions—chi-square reliability may be limited by low expected counts. This interpretation is supported by recent reviews and methodological studies indicating that hair concentrations may reflect a composite of endogenous incorporation during keratinisation and exogenous contributions from atmospheric particulate matter/dust, water, and cosmetic or hygiene practices, with substantial between-person variability and sensitivity to sample preparation and decontamination procedures [12,66,67]. For Sr specifically, experimental cleaning and leaching work has demonstrated separable external and internal Sr pools in hair, indicating that residual exogenous Sr can influence measured values and complicate dietary attribution [68]. More generally, controlled exposure work shows that ambient particulate matter can measurably contaminate hair metal (loid) profiles despite washing, with greater susceptibility for some elements than others [25]. Finally, Spanish total-diet studies from the same era reinforce that ingestion can contribute to baseline exposure for certain elements; in Tarragona, for example, V was detected in the fish/seafood group in a market-basket survey, supporting diet as a plausible contributor to background V intake [69].

Beyond Spanish dietary-habit comparisons, the wider biomonitoring literature suggests that diet–hair relationships for Sr and Ba (often discussed within the alkaline-earth element group) tend to reflect broad dietary habit patterning rather than single “signature” foods. In one scalp-hair study, hair Sr and Ba were positively associated with higher reported frequencies of meat, eggs, fresh vegetables and fruits, and inversely associated with salted vegetables [70]. Similarly, maternal-hair evidence links higher hair Sr/Ba (as alkaline-earth elements) with more frequent consumption of fresh green vegetables, fresh fruit, and meat or fish [71]. By contrast, evidence linking specific food groups to hair vanadium is comparatively limited and inconsistent, and any dietary contribution to hair V is difficult to disentangle from external contact pathways in low-contrast settings. Accordingly, while ingestion may contribute to baseline Sr/Ba (and in some settings V) exposure, the present FFQ data are insufficiently resolved to support food-specific attribution of the intra-urban spatial structure observed in hair Ba–Sr (and the weaker structure for V).

However, because the food-group consumption frequency data were aggregated by age–sex stratum, were not participant-linked, and were not available by residential zone, they are insufficiently resolved to evaluate individual-level nutritional contributions or to test whether diet explains the observed intra-urban spatial gradients in hair Ba and Sr. Moreover, because hair integrates both internal and external inputs, the present FFQ data cannot exclude the possibility that individual dietary preferences contributed to hair Ba or Sr variability in some participants. Accordingly, food intake is interpreted here as descriptive context for plausible ingestion pathways rather than as an explanatory variable, and the observed Ba–Sr spatial structure is discussed only as being compatible with heterogeneous external contact pathways, not as proof of them.

Although diet is a key exposure pathway for several metals and diet–biomarker relationships have been reported in specific settings (e.g., fish consumption and hair mercury in Tarragona), the food-group consumption frequency summaries available in the present study were limited to stratum-level descriptive distributions and were therefore not suitable for diet–hair modelling or for estimating element-specific intake. Consistent with this cautious interpretation, a young-adult cohort (18–22 years) from the same municipality [72] reported little evidence of diet–hair concordance for most elements (with cadmium as an exception), underscoring the limited explanatory power of FFQ-derived intake estimates for inter-individual variability in hair metal concentrations.

### 4.4. Benchmarking Against Spain and Europe: Relative Magnitude and Interpretation

When contextualised against Spanish paediatric hair distributions intended for reference and screening-style interpretation, Alcalá does not appear to exhibit a generalised population-wide elevation across Ba, Sr and V. Instead, the distinguishing feature is distributional structure—particularly the behaviour of the upper tail and the emergence of stronger gradients in adolescence—which is precisely the type of pattern that hair is suited to capture as an indicator of heterogeneous microenvironmental contact [25,26,27].

In children, benchmarking against Spanish paediatric comparators is more consistent with a background regime than with elevated exposure. Children’s Ba and Sr upper tails remain below both the Madrid paediatric benchmark (Ba P95 1.8 µg/g; Sr P95 6.74 µg/g) and the Elche reference compilation (Ba P95 0.901 µg/g; Sr P95 13.37 µg/g), with ER values < 1 for both elements; this indicates that the child distribution is not shifted upwards and that the principal value of the child dataset lies in internal structuring rather than absolute magnitude [26,27]. Notably, despite this background positioning, Ba and Sr show clear sex structuring in children, which is most parsimoniously interpreted as reflecting behavioural and hair-surface determinants (particle retention time, grooming/washing and microenvironmental contact) that modulate exogenous deposition, rather than a municipality-wide source shift acting uniformly on the cohort. The strong Sr–Ba coupling observed in children is compatible with a shared mineral/particle reservoir (resuspended soil/road dust and surface-material contact), supporting interpretation of Ba and Sr as conservative tracers of heterogeneous particulate exposure even when absolute concentrations are modest [23,25].

By contrast, apparent screening exceedance for children’s V can arise under legacy LoD-substitution summaries, but this is precisely the scenario in which inference is most sensitive to left-censoring (79.5% <LoD) and should therefore be treated as provisional until harmonised censored-data re-estimation is applied [24,62]. Finally, spatial inference in children is constrained because recruitment did not cover all municipal zones; therefore, weaker zone structuring in children should not be interpreted as evidence of spatial homogeneity.

For Ba, Alcalá adolescents show an upper-tail enrichment relative to Spanish screening comparators, exceeding the conservative threshold proposed by Ruiz et al. [27] and modestly exceeding the Madrid paediatric upper-tail benchmark reported by Llorente Ballesteros et al. [26]. This pattern is environmentally meaningful because it indicates the presence of a high-exposure subgroup and is consistent with intensified contact with Ba-bearing particulate reservoirs (e.g., road-deposited dust and mechanically generated non-exhaust traffic mixtures) rather than a uniform community-wide shift [26,27]. A comparable interpretation is supported by European hair studies in southern Italy, which show that Ba can vary widely in children and adolescents depending on local environmental context and that upper-tail behaviour can be pronounced even in ostensibly non-contaminated settings, underscoring the sensitivity of hair Ba to microenvironmental differences [28,73]. A particularly informative European comparator is the adolescent survey by Tamburo et al. [60], conducted across contrasted Sicilian microenvironments (11–14 years). In the full dataset (*n* = 943), the median (IQR) concentrations were 1.07 (0.64–1.55) µg/g for Ba, 3.9 (1.7–11) µg/g for Sr, and 0.10 (0.10–0.10) µg/g for V. When set against the Alcalá adolescent medians (Ba 0.287 µg/g; Sr 1.105 µg/g; V 0.011 µg/g; Table 2), the central tendency in Alcalá is lower for each element—by approximately 3.7-fold for Ba, 3.5-fold for Sr and about nine-fold for V. This indicates that the Alcalá adolescent profile is not elevated in absolute terms relative to Mediterranean settings characterised by greater industrial complexity, while remaining compatible with meaningful within-municipality exposure heterogeneity. Tamburo et al. [60] further demonstrated pronounced microenvironmental contrasts consistent with source- and contact-driven variability. In the industrial area, median Ba was 0.9 µg/g in males and 2.0 µg/g in females, and median Sr reached 5.0 µg/g in males and 19 µg/g in females; in the large urban area, median Sr was 3.0 µg/g in males and 8.0 µg/g in females. These site- and sex-stratified medians exceed the corresponding Alcalá central values (Table 1 and Table 2), reinforcing the interpretation that the Alcalá signal is dominated by intra-urban differences in dust- and surface-material contact rather than by the magnitude typically observed in industrial and petrochemical environments. Mechanistically, the prominence of Ba in the Alcalá adolescent upper tail is consistent with contemporary evidence that non-exhaust traffic emissions and resuspension of road-deposited material can dominate near-road particulate mixtures and generate neighbourhood-scale gradients. Ba is widely used as a brake-wear tracer in urban source apportionment, and recent work indicates that resuspended dust together with brake and tyre wear can account for a substantial fraction of traffic-related particulate matter (PM) in urban corridors [22,74]. Accordingly, a municipality-scale Ba gradient in hair is plausibly mediated by differential contact with road-deposited dust and abrasion-derived particles, without requiring a single point source or a uniform population-wide elevation [75]. This profile is consistent with place-based exposure determinants and supports mitigation strategies focused on dust resuspension and non-exhaust sources in higher-burden zones, rather than measures aimed solely at exhaust emissions. Because Ba was not measured in the local topsoils in the present study, future work should quantify Ba in topsoil and road-deposited sediment across the municipal zones to test whether the hair Ba gradient co-varies with geogenic background and traffic-related surface reservoirs, thereby strengthening source attribution.

For Sr, Alcalá remains within the breadth of Spanish paediatric variability and does not exceed the Madrid benchmark, while approaching (but not exceeding) conservative screening-style thresholds in the adolescent upper tail. This is compatible with Sr acting primarily as a tracer of mineral/particle contact rather than a specific hazard signal at environmental levels [27]. European evidence reinforces the plausibility of sex- and context-related heterogeneity in Sr: multi-site adolescent datasets show higher hair Sr in females and substantial spatial variability, consistent with Sr being influenced by both external deposition and the geochemical character of local surface materials [60,76].

For V, adolescents fall well below Spanish screening thresholds and sit within the low-level range expected in general-population contexts, consistent with a background regime rather than an intense and sustained V-enriched emission setting [27,62]. This pattern is also compatible with the well-known source complexity of V, for which soil and environmental levels can reflect mixed controls from parent material and anthropogenic inputs, with local geochemical context modulating whether urban enrichment is detectable [77]. In children, upper-tail screening exceedance can appear when legacy substitution-based summaries are used; however, this is precisely the scenario in which inference is most sensitive to censoring treatment. Given the evidence from Spanish hair monitoring that V can be sparsely detectable and unstable to standard summary statistics in young cohorts, interpretation should emphasise analytical and statistical robustness, prioritising censored-data methods before drawing source or risk conclusions for the children’s V upper tail [24,62]. Under heavy censoring, rank-based correspondence and spatial contrasts can be dominated by a small number of detectable values, so apparent ‘signals’ should be interpreted cautiously until sensitivity analyses confirm robustness [24]. In contrast, marked V elevation in children’s hair has been documented in strong natural source environments (e.g., volcanic influence near Mount Etna), underscoring the context-dependence of hair V as an exposure tracer [78].

The European literature also provides a useful contrast regarding what would be expected under stronger industrial influence. Adolescents residing near petrochemical complexes in Sicily exhibit broader and more coherent multi-element differences in hair relative to control areas, indicating that hair can reflect sustained and spatially coherent industrial signals when present [79]. Against this benchmark, the Alcalá pattern—stronger Ba–Sr structuring with comparatively weak V patterning—supports an interpretation centred on dust/surface-material dynamics and non-exhaust traffic mixtures, rather than a dominant V-rich industrial emission regime affecting the adolescent cohort.

### 4.5. Screening Exceedance and Toxicological Relevance

Ba, Sr and V differ substantially in toxicological potency, target organs and the evidentiary basis for child-health concern. Stable Sr is generally considered of relatively low systemic toxicity at typical environmental exposure levels; however, high exposures may perturb calcium metabolism and skeletal mineralisation, and strontium is toxicologically distinct when present as radioisotopes [34]. Ba has no known biological function and the best-characterised hazards arise from exposure to more soluble Ba compounds that release Ba^2+^, producing acute toxicity with neuromuscular and cardiovascular manifestations mediated, in part, through interference with potassium conductance; nevertheless, environmental Ba is frequently encountered in less soluble particulate forms, and hair does not resolve speciation [35]. V (particularly certain oxidation states and compounds) has a well-established respiratory hazard profile by inhalation, with inflammation and airway effects as key concerns, and recent epidemiological evidence associates environmental V exposure with respiratory and renal endpoints in children [80]. Moreover, recent regulatory toxicology work has re-evaluated vanadium’s systemic hazard profile and derived a parenteral tolerable intake for medical-device applications, providing updated hazard characterisation that complements older profile summaries [38].

In paediatric environmental health, direct inference of hazard from hair concentrations alone is not justified. Nonetheless, a risk-oriented interpretation is supported when (i) distributions intersect published screening thresholds, (ii) coherent spatial gradients point to place-based exposure determinants, and (iii) external toxicological and epidemiological evidence indicates plausible susceptibility at low-to-moderate exposures. Within this framework, the Alcalá results are most appropriately interpreted as indicating spatially structured exposure heterogeneity—strongest for Ba and Sr, and more context-sensitive for V—thereby supporting targeted exposure-reduction priorities in higher-burden microenvironments for susceptible life stages (children and adolescents). Importantly, ER > 1 in this study is interpreted as a flag for exposure prioritisation rather than evidence of toxicity: the most defensible concern is the adolescent Ba upper tail (ER > 1 across comparators), which identifies a higher-contact subgroup potentially influenced by traffic/dust-associated particle mixtures; the borderline Sr exceedance in adolescent females (ER ≳ 1 against the Ruiz limit) is more consistent with mineral/particle tracer behaviour than a primary hazard signal; and the apparent child V exceedance (ER > 1) should be regarded as provisional given heavy left-censoring and the requirement for harmonised censored-data re-estimation before drawing risk inferences. Accordingly, the present interpretation treats hair as an exposure-heterogeneity marker suitable for screening prioritisation, not as a quantitative surrogate of absorbed dose. This ER-based framing therefore reflects screening prioritisation and exposure reduction, not evidence of clinical toxicity or absorbed-dose quantification.

The screening relevance of the Alcalá Ba pattern is strengthened by mechanistic plausibility: non-exhaust traffic emissions (brake and tyre wear, road-surface abrasion) and resuspension of road-deposited dust can dominate near-road PM mixtures and generate neighbourhood-scale gradients, with Ba widely used as a brake-wear tracer in source-apportionment frameworks [22,23,74,75]. Accordingly, an adolescent Ba upper tail intersecting conservative Spanish screening thresholds is most parsimoniously interpreted as reflecting heterogeneous contact with Ba-bearing particulate reservoirs, rather than a community-wide elevation in internal dose, and it supports targeted investigation and exposure-reduction priorities in higher-burden microenvironments.

Several knowledge gaps limit the strength of causal attribution for Ba–Sr–V in paediatric hair and merit targeted investigation. First, the relative contribution of exogenous deposition versus endogenous incorporation remains incompletely resolved for Ba and Sr under real-world urban conditions, and speciation/bioaccessibility cannot be inferred from bulk hair concentrations. Second, the source specificity of V in low-level community settings is uncertain when detectability is limited; resolving this will require harmonised censored-data methods and triangulation with co-occurring combustion tracers and environmental matrices (air PM, indoor dust, road-deposited sediment). Third, municipal-scale inference would be strengthened by integrating geogenic baselines (topsoil Ba/Sr/V) and non-exhaust traffic indicators to test whether hair gradients track surface reservoirs and resuspension dynamics.

### 4.6. Limitations

Several limitations inherent to hair biomonitoring are particularly relevant for Ba–Sr–V. First, external deposition and incomplete removal of particle-associated metals can contribute materially to measured concentrations, potentially inflating upper tails and modest group differences; this is especially pertinent for Ba and Sr, and for settings with sustained particulate exposure where washing may not fully remove fine particles [25,61]. Analytical comparability also depends on harmonised hair preparation and ICP-MS workflows for multi-element determination [81]. Second, information on personal-care practices (e.g., washing frequency, hair length and use of treatments) is often incomplete in retrospective cohorts, limiting causal attribution of sex contrasts. Third, hair does not provide a direct estimate of internal dose, and toxicological inference should therefore be anchored to screening-oriented prioritisation and triangulation with environmental evidence rather than treated as a health-effect biomarker.

Notwithstanding these constraints, the joint evaluation of sex-, age- and intra-urban contrasts provides a coherent exposure narrative in which Ba and Sr behave predominantly as stable particulate/dust tracers with structured heterogeneity, whereas V requires more cautious source-context interpretation. Where feasible, follow-up should prioritise (i) co-located characterisation of outdoor and indoor dust and road-deposited sediment in higher-burden zones, (ii) triangulation with complementary biomarkers less sensitive to external deposition for V and Ba (e.g., urine), and (iii) source-apportionment enhancements for Sr (e.g., isotopic approaches), given the established use of Sr isotope ratios in hair to resolve geographic and geogenic versus dietary contributions [68]. These steps would allow the observed hair patterns to be triangulated against less contamination-prone matrices and co-located environmental reservoirs, strengthening causal inference while retaining the practical value of hair for identifying spatially structured exposure heterogeneity.

## 5. Conclusions

This study establishes a historically informative baseline (April–May 2001) for Ba, Sr and V in paediatric scalp hair from an urban–industrial municipality in central Spain (Alcalá de Henares), and demonstrates the utility of a tracer-oriented interpretation when clinical guidance values for hair are unavailable. The contribution of this work lies in (i) leveraging a legacy paediatric cohort, (ii) applying a compact Ba–Sr–V tracer panel, (iii) integrating contemporaneous residential topsoil information (available for V), and (iv) using a harmonised, censoring-aware analytical/statistical framework that avoids ad hoc substitution and supports transparent, screening-level inference.

Within this framework, Ba and Sr behaved as a coherent conservative pair, exhibiting strong internal co-variation and—most notably—pronounced intra-urban gradients in adolescents. Because no paired topsoil measurements were available for Ba or Sr, these patterns cannot be directly linked to matched environmental reservoirs in the present study. In particular, because site-specific soil-type and zone-level geochemical information for Ba and Sr was not available, it is not possible to distinguish confidently between environmental background differences and variation in exogenous dust contact or anthropogenic redistribution across neighbourhoods. Instead, the observed patterns are interpreted cautiously as being compatible with heterogeneous contact with mineral/road-dust and traffic-influenced surface materials and/or other near-surface exposure microenvironments, rather than as evidence of a municipality-wide elevation in internal dose. Benchmarking against Spanish paediatric hair reference datasets suggests that median Ba and Sr fall within expected national variability, whereas the upper tail for Ba in adolescents appears comparatively elevated in this early-2000s setting. Food-group frequency patterns were broadly concordant with contemporaneous Spanish paediatric surveys; however, because the FFQ data were aggregated and not participant-linked or zone-stratified, they provide descriptive context only and cannot be used to exclude individual dietary contributions or to explain the observed intra-urban Ba–Sr gradients.

In contrast, V showed low hair concentrations, substantial left-censoring, and weak spatial discrimination, consistent with limited environmental contrast in co-located residential topsoils and with wider evidence that hair V is most informative under strong volcanic influence or high-intensity combustion/industrial source regimes. Soil–hair V correspondence was weak overall, with limited support for monotonic coupling under constrained topsoil variability. Sex-stratified analyses suggested differences in direction and magnitude: adolescent girls showed moderate correspondence, whereas adolescent boys showed a modest inverse, non-significant trend (*ρ* = −0.310; *p* = 0.101; *n* = 29). The inverse, non-significant trend in adolescent boys (*n* = 29) should be interpreted cautiously given limited power and residual left-censoring. Behavioural proxy mismatch is plausible in adolescence, as time–activity patterns and contact with non-residential surfaces (e.g., sports grounds, commuting corridors) may weaken or invert correspondence with co-located residential topsoil when environmental contrast is small.

Three implications follow. First, the tight coherence and neighbourhood-scale gradients of Ba/Sr suggest that these elements may serve as practical screening-level tracers of dust- and traffic-influenced surface materials in adolescent hair under heterogeneous urban microenvironments, although this interpretation requires confirmation with paired environmental matrices. Second, the weaker spatial structure for V underscores the importance of matrix triangulation and consistent censored-data handling in low-level community settings. Third, municipal-scale source attribution would be materially strengthened by adding paired environmental matrices for Ba and Sr (e.g., topsoil, road-deposited sediment, indoor dust) and, where feasible, by incorporating isotopic, speciation, or microanalytical approaches capable of better distinguishing exogenous particulate retention from keratin-associated incorporation. Overall, these findings are screening- and source-oriented and should not be interpreted as direct estimates of internal dose or clinical risk based on hair concentrations alone.

## Figures and Tables

**Table 1:** Concentrations of metals and metalloids (µg/g) in hair of children from Alcalá de Henares, Spain.

Element	Group	*N*	% <LoD	LoD	A.M.	G.M.	Median	IQR	P95	CI-PP95	Range	*p*-Value (Sex)
**Ba**	**Total**	74	38.3	0.124	0.273 ± 0.191	0.175	0.193	0.124, 0.350	0.629	0.447–0.687	0.136–1.290	0.000004 ***
	**Boys**	21	58.0	0.124	0.154 ± 0.141	0.106	0.108	0.062, 0.193	0.485	0.167–0.515	0.136–0.687	
	**Girls**	53	24.3	0.124	0.333 ± 0.209	0.245	0.271	0.153, 0.421	0.717	0.533–0.848	0.153–1.290	
**Sr**	**Total**	92	23.3	0.187	0.680 ± 0.654	0.413	0.412	0.195, 0.894	2.155	1.183–2.509	0.191–3.366	6.78 × 10^−10^ ***
	**Boys**	28	44.0	0.187	0.367 ± 0.476	0.205	0.197	0.187, 0.377	0.758	0.377–0.958	0.191–3.366	
	**Girls**	64	8.6	0.187	0.908 ± 0.672	0.670	0.688	0.359, 1.162	2.441	1.770–2.646	0.228–2.743	
**V** **^†^**	**Total**	24	79.5	0.02	0.44 ± 0.41	0.32	0.27	0.16, 0.50	1.21	1.04–1.37	0.12–1.77	NS
	**Boys**	13	72.3	0.02	0.48 ± 0.49	0.35	0.27	0.24, 0.44	1.47	1.20–1.73	0.13–1.77	
	**Girls**	11	84.3	0.02	0.38 ± 0.30	0.29	0.27	0.14, 0.58	0.86	0.68–1.04	0.12–0.89	
**V**	**Total**	31	74.2	0.02	0.274 ± 24.083	0.003	0.003	0.0004, 0.0235	0.428	0.165–1.109	0.023–1.026	0.0243
	**Boys**	18	64.0	0.02	0.110 ± 0.230	0.008	0.013	0.003, 0.094	0.567	0.020–0.687	0.023–1.026	
	**Girls**	13	81.4	0.02	/	0.0014	/	/	0.222	0.328 ^††^	0.023–0.706	

*N* = number of samples above LoD; % ≤ LoD = percentage below limit of detection; A.M. = arithmetic mean (results are presented as mean values ± S.D.); G.M. = geometric mean; IQR = interquartile range (P25, P75); P95 = 95th percentile; CI-PP95 = 95% confidence interval for 95PP (population percentile); V^†^ indicates historical vanadium data from Peña-Fernández [29], whereas V without the dagger corresponds to the present study analysis; ^††^ 97.5th percentile; / = the data does not provide enough information to determine this parameter. * *p* < 0.05; ** *p* < 0.01; *** *p* < 0.001.

**Table 2:** Concentrations of metals and metalloids (µg/g) in hair of adolescents from Alcalá de Henares, Spain.

Element	Group	*N*	% <LoD	LoD	A.M.	G.M.	Median	IQR	P95	CI-PP95	Range	*p*-Value (Sex)	*p*-Value (Age)
**Ba**	**Total**	63	35.1	0.153	0.671 ± 0.750	0.286	0.287	0.153, 0.948	2.061	1.679–2.668	0.155–3.469	0.235	0.0014
	**Boys**	12	58.6	0.153	0.847 ± 0.921	0.113	0.369	0.197, 1.458	2.425	0.153–3.148	0.287–3.469		
	**Girls**	51	25.0	0.153	0.619 ± 0.645	0.340	0.323	0.153, 0.777	2.004	1.099–2.210	0.155–3.050		
**Sr**	**Total**	61	37.1	0.434	1.780 ± 1.904	0.808	1.105	0.434, 2.390	4.995	3.454–6.342	0.513–13.166	0.006 **	1.67 × 10^−5^
	**Boys**	11	62.1	0.434	1.099 ± 0.981	0.291	0.668	0.397, 1.766	2.786	0.434–3.273	0.513–3.861		
	**Girls**	50	26.5	0.434	2.079 ± 2.119	1.105	1.240	0.434, 3.066	6.023	3.609–7.107	0.578–13.166		
**V**	**Total**	47	51.5	0.011	0.017 ± 0.021	0.011	0.011	0.006, 0.020	0.052	0.037–0.072	0.011–0.166	0.004 **	0.365
	**Boys**	7	75.9	0.011	0.012 ± 0.033	0.003	0.001	0.0002, 0.0050	0.057	0.011–0.080	0.012–0.166		
	**Girls**	40	41.2	0.011	0.020 ± 0.016	0.013	0.013	0.011, 0.021	0.053	0.025–0.077	0.011–0.091		

*N* = number of samples above LoD; % ≤ LoD = percentage below limit of detection; A.M. = arithmetic mean (results are presented as mean values ± S.D.); G.M. = geometric mean; IQR = interquartile range (P25, P75); P95 = 95th percentile; CI-PP95 = 95% confidence interval for 95PP (population percentile); * *p* < 0.05; ** *p* < 0.01; *** *p* < 0.001.

**Table 3:** Spatial (zone) differences in hair element concentrations (µg/g) in children (Alcalá de Henares).

Element	Zone	*N*	KM Mean	LCL Mean	*p*-Value
**Ba**	1	24	0.361 ± 0.202	0.273	0.053
	2	60	0.273 ± 0.199	0.221	
	4	36	0.251 ± 0.112	0.212	
**Sr**	1	24	0.619 ± 0.423	0.435	0.736
	2	60	0.618 ± 0.574	0.468	
	4	36	0.829 ± 0.842	0.539	
**V** **^†^**	1	24	0.32 ± 0.30	0.15	NS
	2	60	0.44 ± 0.52	0.25	
	4	36	0.49 ± 0.35	0.31	
**V**	1	24	0.059 ± 0.078	0.024	0.139
	2	60	0.082 ± 0.133	0.045	
	4	36	0.131 ± 0.232	0.049	

Zones correspond to the residential-area classification described in Section 2 and Figure 1. *N* = number of participants in each zone. For variables with left-censored observations (<LoD), zone-level central tendency was estimated using the Kaplan–Meier mean (KM Mean) (reported as mean ± dispersion as shown in the table); LCL Mean = lower 95% confidence limit of the KM mean. *p*-value denotes the overall comparison among zones using the Peto–Peto (modified Gehan–Wilcoxon) test for censored variables. No statistically significant overall zone effect was observed for the variables shown in this table; therefore, no post hoc pairwise groupings are displayed. V^†^ indicates historical vanadium data from Peña-Fernández [29], whereas V without the dagger corresponds to the present study analysis; results are presented as arithmetic mean values ± S.D.

**Table 4:** Spatial (zone) differences in hair element concentrations (µg/g) in adolescents (Alcalá de Henares).

Element	Zone	*N*	KM Mean	LCL Mean	*p*-Value
**Ba**	1	36	0.297 ± 0.357	0.172 ^a^	2.66 × 10^−18^ ***
	2	20	0.537 ± 0.581	0.254 ^a^	
	3	24	0.535 ± 0.424	0.352 ^b^	
	4	17	1.872 ± 0.645	1.529 ^c^	
**Sr**	1	36	0.989 ± 0.959	0.652 ^a^	2.61 × 10^−9^ ***
	2	20	1.489 ± 1.252	0.875 ^a^	
	3	24	1.865 ± 1.299	1.302 ^b^	
	4	17	3.832 ± 2.859	2.314 ^c^	
**V**	1	36	0.021 ± 0.028	0.011	0.162
	2	20	0.017 ± 0.006	0.014	
	3	24	0.022 ± 0.020	0.014	
	4	17	0.023 ± 0.015	0.014	

Zones correspond to the residential-area classification described in Section 2 and Figure 1. *N* = number of participants in each zone. For variables with left-censored observations (<LoD), zone-level central tendency was estimated using the Kaplan–Meier mean (KM Mean) (reported as mean ± dispersion as shown in the table); LCL Mean = lower 95% confidence limit of the KM mean. *p*-value denotes the overall comparison among zones using the Peto–Peto (modified Gehan–Wilcoxon) test for censored variables. Where the overall test was significant, post hoc pairwise comparisons were performed; different superscript letters (a, b, c) indicate statistically significant differences between zones (same letter = not significantly different). *p*-values from multiple comparisons were adjusted using the Benjamini–Hochberg false discovery rate (FDR) procedure. Asterisks denote significance levels: * *p* < 0.05; ** *p* < 0.01; *** *p* < 0.001.

## Data Availability

Data for this article is available at Open Science Framework (OSF) at: https://osf.io/69v78/overview?view_only=f05b8ba753324acc9ae8f293231d37a8 (accessed on 14 February 2026).

## Supplementary Materials

The following supporting information can be downloaded at: https://www.mdpi.com/article/10.3390/toxics14030268/s1, Table S1: Food-group consumption frequency in children and adolescents, by sex (%); Table S2: Exploratory age–sex group contrasts in FFQ consumption frequency (Pearson chi-square test) with Benjamini–Hochberg FDR adjustment, by food group.

## References

[1] Xue J., Zartarian V., Moya J., Freeman N., Beamer P., Black K., Tulve N., Shalat S. (2007). A meta-analysis of children’s hand-to-mouth frequency data for estimating nondietary ingestion exposure. Risk Anal..

[2] Moya J., Phillips L. (2014). A review of soil and dust ingestion studies for children. J. Expo. Sci. Environ. Epidemiol..

[3] Martínez-Morata I., Sobel M., Tellez-Plaza M., Navas-Acien A., Howe C.G., Sanchez T.R. (2023). A State-of-the-Science Review on Metal Biomarkers. Curr. Environ. Health Rep..

[4] Werkenthin M., Kluge B., Wessolek G. (2014). Metals in European roadside soils and soil solution—A review. Environ. Pollut..

[5] Binner H., Sullivan T., Jansen M.A.K., McNamara M.E. (2023). Metals in urban soils of Europe: A systematic review. Sci. Total Environ..

[6] Lucchini R.G., Guazzetti S., Renzetti S., Conversano M., Cagna G., Fedrighi C., Giorgino A., Peli M., Placidi D., Zoni S. (2019). Neurocognitive impact of metal exposure and social stressors among schoolchildren in Taranto, Italy. Environ. Health Glob. Access Sci. Source.

[7] Butler L., Gennings C., Peli M., Borgese L., Placidi D., Zimmerman N., Hsu H.L., Coull B.A., Wright R.O., Smith D.R. (2019). Assessing the contributions of metals in environmental media to exposure biomarkers in a region of ferroalloy industry. J. Expo. Sci. Environ. Epidemiol..

[8] Junqué E., Tardón A., Fernandez-Somoano A., Grimalt J.O. (2022). Environmental and dietary determinants of metal exposure in four-year-old children from a cohort located in an industrial area (Asturias, Northern Spain). Environ. Res..

[9] Roca M., Sánchez A., Pérez R., Pardo O., Yusà V. (2016). Biomonitoring of 20 elements in urine of children. Levels and predictors of exposure. Chemosphere.

[10] Pérez R., Doménech E., Conchado A., Sanchez A., Coscollà C., Yusà V. (2018). Influence of diet in urinary levels of metals in a biomonitoring study of a child population of the Valencian region (Spain). Sci. Total Environ..

[11] Sáez C., Sánchez A., Molina Y., Suelves T., Llop S., Soler-Blasco R., Dualde P., Aguirre M.Á., Canals A., Coscollà C. (2026). Biomonitoring of Metals in the Urine of Children from the Valencian Region (Spain): Levels, Predictors of Exposure, and Risk Assessment. Expo. Health.

[12] Hsu J.F., Hsu J.Y., Hsiao P.Z., Chou T.C., Liao P.C. (2024). Hair specimens in exposome-health research: Opportunities, challenges, and applications. TrAC Trends Anal. Chem..

[13] Lallmahomed A., Mercier F., Costet N., Fillol C., Bonvallot N., Le Bot B. (2024). Characterization of organic contaminants in hair for biomonitoring purposes. Environ. Int..

[14] Apeagyei E., Bank M.S., Spengler J.D. (2011). Distribution of heavy metals in road dust along an urban-rural gradient in Massachusetts. Atmos. Environ..

[15] Wiseman C.L.S., Levesque C., Rasmussen P.E. (2021). Characterizing the sources, concentrations and resuspension potential of metals and metalloids in the thoracic fraction of urban road dust. Sci. Total Environ..

[16] Reimann C., Birke M., Demetriades A., Filzmoser P., O’Connor P. (2014). Chemistry of Europe’s Agricultural Soils. Part A: Methodology and Interpretation of the GEMAS Data Set.

[17] Martín-Méndez I., Prazeres C., Locutura J., Batista M.J., Bel-lan A. (2015). Análisis factorial de datos geoquímicos de suelos de la Península Ibérica, derivados del Proyecto GEMAS (Eurogeosurveys) [Factor analysis of geochemical soil data from the Iberian Peninsula derived from the GEMAS Project (EuroGeoSurveys)]. Comun. Geológicas.

[18] Wnuk E. (2023). Mobility, bioavailability, and toxicity of vanadium regulated by physicochemical and biological properties of the soil. J. Soil Sci. Plant Nutr..

[19] Adewumi A.J., Ogundele O.D. (2024). Hidden hazards in urban soils: A meta-analysis review of global heavy metal contamination (2010–2022), sources and its Ecological and health consequences. Sustain. Environ..

[20] Tyszka R., Pędziwiatr A., Pietranik A., Kierczak J., Ettler V., Mihaljevič M., Zieliński G. (2024). A long-term perspective on coal combustion solid waste interacting with urban soil. Appl. Geochem..

[21] Peña-Fernández A., Lobo-Bedmar M.C., González-Muñoz M.J. (2015). Annual and seasonal variability of metals and metalloids in urban and industrial soils in Alcalá de Henares (Spain). Environ. Res..

[22] Hicks W., Beevers S., Tremper A.H., Stewart G., Priestman M., Kelly F.J., Lanoisellé M., Lowry D., Green D.C. (2021). Quantification of Non-Exhaust Particulate Matter Traffic Emissions and the Impact of COVID-19 Lockdown at London Marylebone Road. Atmosphere.

[23] Fussell J.C., Franklin M., Green D.C., Gustafsson M., Harrison R.M., Hicks W., Kelly F.J., Kishta F., Miller M.R., Mudway I.S. (2022). A Review of Road Traffic-Derived Non-Exhaust Particles: Emissions, Physicochemical Characteristics, Health Risks, and Mitigation Measures. Environ. Sci. Technol..

[24] Helsel D.R. (2011). Statistics for Censored Environmental Data Using Minitab and R.

[25] Ren M., Yan L., Pang Y., Jia X., Huang J., Shen G., Cheng H., Wang X., Pan B., Li Z. (2020). External interference from ambient air pollution on using hair metal(loid)s for biomarker-based exposure assessment. Environ. Int..

[26] Llorente Ballesteros M.T., Navarro Serrano I., Izquierdo Álvarez S. (2017). Reference levels of trace elements in hair samples from children and adolescents in Madrid, Spain. J. Trace Elem. Med. Biol. Organ Soc. Miner. Trace Elem. (GMS).

[27] Ruiz R., Estevan C., Estévez J., Alcaide C., Sogorb M.A., Vilanova E. (2023). Reference Values on Children’s Hair for 28 Elements (Heavy Metals and Essential Elements) Based on a Pilot Study in a Representative Non-Contaminated Local Area. Int. J. Mol. Sci..

[28] Dongarrà G., Lombardo M., Tamburo E., Varrica D., Cibella F., Cuttitta G. (2011). Concentration and reference interval of trace elements in human hair from students living in Palermo, Sicily (Italy). Environ. Toxicol. Pharmacol..

[29] Peña-Fernández A. (2011). Presencia y Distribución Medioambiental de Metales Pesados y Metaloides en Alcalá de Henares, Madrid. Evaluación del Riesgo Para la Población y Biomonitorización de la Población Escolar. Doctoral Thesis.

[30] Eastman R.R., Jursa T.P., Benedetti C., Lucchini R.G., Smith D.R. (2013). Hair as a biomarker of environmental manganese exposure. Environ. Sci. Technol..

[31] Rodrigues J.L., Batista B.L., Nunes J.A., Passos C.J., Barbosa F. (2008). Evaluation of the use of human hair for biomonitoring the deficiency of essential and exposure to toxic elements. Sci. Total Environ..

[32] LeBeau M.A., Montgomery M.A., Brewer J.D. (2011). The role of variations in growth rate and sample collection on interpreting results of segmental analyses of hair. Forensic Sci. Int..

[33] Favretto D., Cooper G., Andraus M., Sporkert F., Agius R., Appenzeller B., Baumgartner M., Binz T., Cirimele V., Kronstrand R. (2023). The Society of Hair Testing consensus on general recommendations for hair testing and drugs of abuse testing in hair. Drug Test. Anal..

[34] Ru X., Yang L., Shen G., Wang K., Xu Z., Bian W., Zhu W., Guo Y. (2024). Microelement strontium and human health: Comprehensive analysis of the role in inflammation and non-communicable diseases (NCDs). Front. Chem..

[35] Peana M., Medici S., Dadar M., Zoroddu M.A., Pelucelli A., Chasapis C.T., Bjørklund G. (2021). Environmental barium: Potential exposure and health-hazards. Arch. Toxicol..

[36] Perelló G., Vicente E., Castell V., Llobet J.M., Nadal M., Domingo J.L. (2015). Dietary intake of trace elements by the population of Catalonia (Spain): Results from a total diet study. Food Addit. Contam. Part A Chem. Anal. Control Expo. Risk Assess..

[37] Ścibior A., Pietrzyk Ł., Plewa Z., Skiba A. (2020). Vanadium: Risks and possible benefits in the light of a comprehensive overview of its pharmacotoxicological mechanisms and multi-applications with a summary of further research trends. J. Trace Elem. Med. Biol. Organ Soc. Miner. Trace Elem. (GMS).

[38] Laupheimer C.E., Kolianchuk Y., FitzGerald R.E., Wilks M.F., Jaksch A. (2025). Toxicological evaluation of vanadium and derivation of a parenteral tolerable intake value for medical devices. Regul. Toxicol. Pharmacol. RTP.

[39] Martin-Moreno J.M., Boyle P., Gorgojo L., Maisonneuve P., Fernandez-Rodriguez J.C., Salvini S., Willett W.C. (1993). Development and validation of a food frequency questionnaire in Spain. Int. J. Epidemiol..

[40] Granero S., Llobet J.M., Schuhmacher M., Corbella J., Domingo J.L. (1998). Biological monitoring of environmental pollution and human exposure to metals in Tarragona, Spain. I. Levels in hair of school children. Trace Elem. Electrolytes.

[41] Llobet J.M., Granero S., Schuhmacher M., Corbella J., Domingo J.L. (1998). Biological monitoring of environmental pollution and human exposure to metals in Tarragona, Spain. IV. Estimation of the dietary intake. Trace Elem. Electrolytes.

[42] Capdevila F., Llop D., Guillén N., Luque V., Pérez S., Sellés V., Fernández-Ballart J., Martí-Henneberg C. (2000). Consumo, hábitos alimentarios y estado nutricional de la población de Reus (X): Evolución de la ingestión alimentaria y de la contribución de los macronutrientes al aporte energético (1983–1999), según edad y sexo. Med. Clin..

[43] Agresti A. (2002). Categorical Data Analysis.

[44] Schuhmacher M., Bellés M., Rico A., Domingo J.L., Corbella J. (1996). Impact of reduction of lead in gasoline on the blood and hair lead levels in the population of Tarragona Province, Spain, 1990–1995. Sci. Total Environ..

[45] Helsel D. (2010). Much ado about next to nothing: Incorporating nondetects in science. Ann. Occup. Hyg..

[46] Shoari N., Dubé J.S. (2018). Toward improved analysis of concentration data: Embracing nondetects. Environ. Toxicol. Chem..

[47] Klein J.P., Moeschberger M.L. (2003). Survival Analysis: Techniques for Censored and Truncated Data.

[48] Lee L. (2025). NADA: Nondetects and Data Analysis for Environmental Data; R Package Version 1.6-1.2. https://cran.r-project.org/web/packages/NADA/index.html.

[49] Poulsen O.M., Holst E., Christensen J.M. (1997). Calculation and application of coverage intervals for biological reference values. Pure Appl. Chem..

[50] Julian P., Helsel D., Lee L. (2025). NADA2: Data Analysis for Censored Environmental Data; R Package Version 2.0.1. https://cran.r-project.org/web/packages/NADA2.

[51] R Core Team (2026). R: A Language and Environment for Statistical Computing.

[52] Peto R., Peto J. (1972). Asymptotically efficient rank invariant test procedures. J. R. Stat. Soc. Ser. A (Gen.).

[53] Schulz C., Angerer J., Ewers U., Kolossa-Gehring M. (2007). The German Human Biomonitoring Commission. Int. J. Hyg. Environ. Health.

[54] Helsel D.R. (2006). Fabricating data: How substituting values for nondetects can ruin results, and what can be done about it. Chemosphere.

[55] Hladik M.L., Markus A., Helsel D., Nowell L.H., Polesello S., Rüdel H., Szabo D., Wilson I. (2024). Evaluating the reliability of environmental concentration data to characterize exposure in environmental risk assessments. Integr. Environ. Assess. Manag..

[56] Skröder H., Kippler M., Nermell B., Tofail F., Levi M., Rahman S.M., Raqib R., Vahter M. (2017). Major Limitations in Using Element Concentrations in Hair as Biomarkers of Exposure to Toxic and Essential Trace Elements in Children. Environ. Health Perspect..

[57] Zare Jeddi M., Galea K.S., Ashley-Martin J., Nassif J., Pollock T., Poddalgoda D., Kasiotis K.M., Machera K., Koch H.M., López M.E. (2025). Guidance on minimum information requirements (MIR) from designing to reporting human biomonitoring (HBM). Environ. Int..

[58] Błaszczyk-Altman M., Semla-Kurzawa M., Grzyb K., Kiwacka A., Kucharska K., Massanyi P., Stawarz R., Binkowski L.J. (2025). Implications of air pollution and cosmetic regimens on metal concentrations in women’s hair from Poland. Environ. Sci. Eur..

[59] Vahter M., Akesson A., Lidén C., Ceccatelli S., Berglund M. (2007). Gender differences in the disposition and toxicity of metals. Environ. Res..

[60] Tamburo E., Varrica D., Dongarrà G. (2016). Gender as a key factor in trace metal and metalloid content of human scalp hair. A multi-site study. Sci. Total Environ..

[61] Christensen J.R., LaBine G.O. (2024). Microchemistry of Single Hair Strands Below and Above the Scalp: Impacts of External Contamination on Cuticle and Cortex Layers. Biol. Trace Elem. Res..

[62] Esplugas R., Mari M., Marquès M., Schuhmacher M., Domingo J.L., Nadal M. (2019). Biomonitoring of Trace Elements in Hair of Schoolchildren Living Near a Hazardous Waste Incinerator—A 20 Years Follow-Up. Toxics.

[63] Serra-Majem L., García-Closas R., Ribas L., Pérez-Rodrigo C., Aranceta J. (2001). Food patterns of Spanish schoolchildren and adolescents: The enKid Study. Public Health Nutr..

[64] Serra-Majem L., Ribas L., Ngo J., Ortega R.M., García A., Pérez-Rodrigo C., Aranceta J. (2004). Food, youth and the Mediterranean diet in Spain. Development of KIDMED, Mediterranean Diet Quality Index in children and adolescents. Public Health Nutr..

[65] Fernández San Juan P.M. (2006). Dietary habits and nutritional status of school aged children in Spain. Nutr. Hosp..

[66] David G.K., Hunter A.H., Moromizato K.H., Allen C.M., Wheatley R., von Hippel F.A., Niehaus A.C., Wilson R.S. (2023). Pre-cleaning of hair is not beneficial in LA-ICP-MS studies of chronic metal exposure. PLoS ONE.

[67] Adav S.S., Ng K.W. (2025). The multifaceted role of hair as a biospecimen: Recent advances in precision medicine and forensic science. Exp. Mol. Med..

[68] Tipple B.J., Valenzuela L.O., Chau T.H., Hu L., Bataille C.P., Chesson L.A., Ehleringer J.R. (2019). Strontium isotope ratios of human hair from the United States: Patterns and aberrations. Rapid Commun. Mass Spectrom. RCM.

[69] Bocio A., Nadal M., Domingo J.L. (2005). Human exposure to metals through the diet in Tarragona, Spain: Temporal trend. Biol. Trace Elem. Res..

[70] Wang B., Yan L., Sun Y., Yan J., Lu Q., Zhang J., Li Z. (2017). Alkaline-earth elements of scalp hair and presence of hypertension in housewives: A perspective of chronic effect. Chemosphere.

[71] Li Z., Wang B., Huo W., Liu Y., Zhu Y., Xie J., Li Z., Ren A. (2017). Are concentrations of alkaline earth elements in maternal hair associated with risk of neural tube defects?. Sci. Total Environ..

[72] González-Muñoz M.J., Peña A., Meseguer I. (2008). Monitoring heavy metal contents in food and hair in a sample of young Spanish subjects. Food Chem. Toxicol. Int. J. Publ. Br. Ind. Biol. Res. Assoc..

[73] Dongarrà G., Varrica D., Tamburo E., D’Andrea D. (2012). Trace elements in scalp hair of children living in differing environmental contexts in Sicily (Italy). Environ. Toxicol. Pharmacol..

[74] Chen L.A., Wang X., Lopez B., Wu G., Ho S.S.H., Chow J.C., Watson J.G., Yao Q., Yoon S., Jung H. (2023). Contributions of non-tailpipe emissions to near-road PM_2.5_ and PM_10_: A chemical mass balance study. Environ. Pollut..

[75] He C., Jiang W., Xiao Q., Xing C., Yuan D., Lu R., Wu W. (2024). A review of non-exhaust emissions on pavement area: Sources, compositions, evaluation and mitigation. J. Traffic Transp. Eng. (Engl. Ed.).

[76] Font L., Van Der Peijl G., Van Wetten I., Vroon P., Van Der Wagt B., Davies G. (2012). Strontium and lead isotope ratios in human hair: Investigating a potential tool for determining recent human geographical movements. J. Anal. At. Spectrom..

[77] Guagliardi I., Cicchella D., De Rosa R., Ricca N., Buttafuoco G. (2018). Geochemical sources of vanadium in soils: Evidences in a southern Italy area. J. Geochem. Explor..

[78] Varrica D., Tamburo E., Dongarrà G., Sposito F. (2014). Trace elements in scalp hair of children chronically exposed to volcanic activity (Mt. Etna, Italy). Sci. Total Environ..

[79] Varrica D., Tamburo E., Alaimo M.G. (2022). Levels of trace elements in human hair samples of adolescents living near petrochemical plants. Environ. Geochem. Health.

[80] Rojas-Lima E., Ortega-Romero M., Aztatzi-Aguilar O.G., Rubio-Gutiérrez J.C., Narváez-Morales J., Esparza-García M., Méndez-Hernández P., Medeiros M., Barbier O.C. (2025). Vanadium exposure and kidney markers in a pediatric population: A cross-sectional study. Pediatr. Nephrol..

[81] Runkel A.A., Jagodic Hudobivnik M., Živković I., Klemenčič P., Mazej D., Horvat M. (2026). Optimisation of A Sample Preparation Method for the Determination of Multi-Elemental Compositions in Human Hair By Triple Quadrupole ICP-MS Analysis. Biol. Trace Elem. Res..

