# Supplementary material for: Ba–Sr–V as Geogenic and Traffic Tracers in Paediatric Hair from Urban–Industrial Spain, with Co-Located Topsoil Vanadium
Source: Toxics. 2026 Mar 19;14(3):268. doi: 10.3390/toxics14030268 (PMC13029910; doi:10.3390/toxics14030268)
Supplement: Supplementary file 1 [file toxics-14-00268-s001.zip › Supplementary tables_diet.pdf]

Table S1. Food-group consumption frequency in children and adolescents, by sex (%).

		Food group	NR (%)	Never (%)	<2/week (%)	3-4/week (%)	≥5/week (%)	≥3/week (excl. NR, %)
Children	Boys	Red meat	6.7	13.6	52.5	27.1	0.0	29.0
	Girls		5.5	6.8	63.0	24.6	0.0	26.0
Adolescents	Male		4.8	4.8	66.7	23.8	0.0	25.0
	Female		1.8	10.5	57.9	29.8	0.0	30.4
Children	Boys	Pork	6.8	0.0	66.1	27.1	0.0	29.1
	Girls		5.5	6.8	65.7	21.9	0.0	23.2
Adolescents	Male		7.1	7.1	57.1	26.2	2.4	30.8
	Female		5.3	1.8	64.9	26.3	1.8	29.6
Children	Boys	Chicken/poultry	1.7	0.0	30.5	67.8	0.0	69.0
	Girls		1.4	0.0	32.9	63.0	2.7	66.6
Adolescents	Male		4.8	0.0	21.4	66.7	7.1	77.5
	Female		5.3	0.0	52.6	36.8	5.3	44.4
Children	Boys		1.7	3.4	47.4	39.0	8.5	48.3

	Girls	Processed meats (cured meats/sausages)	2.7	12.3	47.9	30.1	6.8	37.9
Adolescents	Male		2.4	4.8	21.4	50.0	21.4	73.2
	Female		3.5	8.8	47.4	24.6	15.8	41.8
Children	Boys	Fish	1.7	3.4	30.5	61.0	3.4	65.5
	Girls		1.4	2.7	43.8	49.3	2.7	52.7
Adolescents	Male		0.0	7.1	52.4	35.7	4.8	40.5
	Female		0.0	5.3	43.9	43.9	7.0	50.9
Children	Boys	Vegetables	1.7	6.8	30.5	33.4	27.1	61.5
	Girls		1.4	5.5	23.3	45.2	24.6	70.8
Adolescents	Male		0.0	7.1	16.7	40.5	35.7	76.2
	Female		0.0	8.8	22.8	42.1	26.3	68.4
Children	Boys	Rice/pasta	0.0	0.0	28.8	67.8	3.4	71.2
	Girls		0.0	1.4	35.6	54.5	8.2	62.7
Adolescents	Male		0.0	0.0	38.1	50.0	11.9	61.9
	Female		3.5	0.0	42.1	47.4	7.0	56.4
Children	Boys	Legumes	0.0	0.0	47.4	49.1	3.4	52.5
	Girls		2.7	1.4	53.4	39.7	2.7	43.6

Adolescents	Male		0.0	2.4	45.2	45.2	7.1	52.4
	Female		1.8	0.0	54.4	43.9	0.0	44.6
Children	Boys	Bread	0.0	1.7	11.9	11.9	74.6	86.5
	Girls		0.0	2.7	12.3	9.6	75.3	84.9
Adolescents	Male		0.0	4.8	0.0	14.3	81.0	95.2
	Female		0.0	3.5	10.5	21.1	64.9	86.0
Children	Boys	Fruit	1.7	1.7	11.9	20.3	64.4	86.2
	Girls		0.0	5.5	5.5	24.6	64.4	89.0
Adolescents	Male		0.0	9.5	21.4	28.6	40.5	69.0
	Female		0.0	3.5	15.8	21.1	59.6	80.7
Children	Boys	Milk	0.0	0.0	0.0	6.8	93.2	100.0
	Girls		0.0	1.4	0.0	8.2	90.4	98.6
Adolescents	Male		2.4	2.4	0.0	11.9	83.3	97.6
	Female		0.0	5.3	5.3	8.8	80.7	89.5
Children	Boys	Yoghurt	1.7	0.0	5.1	20.3	72.9	94.8
	Girls		1.4	0.0	12.3	9.6	76.7	87.5
Adolescents	Male		0.0	2.4	14.3	30.9	52.4	83.3

	Female		0.0	1.8	24.6	43.9	29.8	73.7
Children	Boys	Eggs	3.4	0.0	67.8	27.1	1.7	29.8
	Girls		0.0	0.0	64.4	34.2	1.4	35.6
Adolescents	Male		0.0	2.4	52.4	45.2	0.0	45.2
	Female		0.0	5.3	61.4	31.6	1.8	33.3
Children	Boys	Canned foods	5.1	42.4	50.8	1.7	0.0	1.8
	Girls		0.0	42.5	53.4	2.7	1.4	4.1
Adolescents	Male		2.4	33.3	45.2	19.1	0.0	19.5
	Female		7.0	24.6	64.9	1.8	1.8	3.8

Table S2. Exploratory age–sex group contrasts in FFQ consumption frequency (Pearson chi-square test) with Benjamini–Hochberg FDR adjustment, by food group.

Food group	Children overall ≥3/week (%, excl. NR)	Adolescents overall ≥3/week (%, excl. NR)	Pearson χ^2 (df)	*p*-value	FDR *q*-value (BH)	*N* valid
Red meat	27.4	28.7	3.967 (6)	0.681	0.733	206
Pork	25.7	30.8	6.783 (6)	0.341	0.597	204
Chicken/poultry	67.8	53.8	12.952 (3)	**0.005**	**0.035**	210
Processed meats (cured meats/sausages)	41.9	52.1	14.227 (6)	**0.027**	0.126	212
Fish	57.6	48.5	5.541 (6)	0.477	0.721	216
Vegetables	67.8	71.1	2.632 (6)	0.853	0.853	215
Rice/pasta	66.7	57.9	5.227 (6)	0.515	0.721	215
Legumes	47.5	46.9	4.505 (6)	0.609	0.733	214
Bread	85.8	88.7	4.149 (6)	0.657	0.733	216
Fruit	87.4	77.3	9.256 (6)	0.160	0.350	216

Milk	99.2	91.7	13.377 (6)	**0.037**	0.130	218
Yoghurt	90.7	76.3	12.166 (6)	0.058	0.162	216
Eggs	33.1	36.1	8.966 (6)	0.175	0.350	216
Canned foods	3.4	7.7	19.371 (6)	**0.004**	**0.035**	209

% ≥3/week (excl. NR) calculated among respondents with non-missing FFQ data for that item.
